# Systematic revision of the trans-Bassian moriomorphine genus *Theprisa* Moore (Coleoptera, Carabidae)

**DOI:** 10.3897/zookeys.1044.62335

**Published:** 2021-06-16

**Authors:** James K. Liebherr, Nick Porch, Matthew Shaw, Bronte E. Sinclair, David R. Maddison

**Affiliations:** 1 Department of Entomology, John H. and Anna B. Comstock Hall, 129 Garden Ave., Cornell University, Ithaca, NY 14853-2601, USA Cornell University Ithaca United States of America; 2 School of Life & Environmental Sciences & Centre for Integrated Ecology, Deakin University, Geelong, VIC 3216, Australia Deakin University Geelong Australia; 3 South Australian Museum, South Terrace, Adelaide, SA 5000, Australia South Australian Museum Adelaide Australia; 4 Australian National Insect Collection, Building 101, Clunies Ross St., Black Mountain, ACT 2601, Australia Australian National Insect Collection Black Mountain Australia; 5 Department of Integrative Biology, Oregon State University, Corvallis, OR 97331, USA Oregon State University Corvallis United States of America

**Keywords:** Allopatric speciation, area of endemism, biogeography, generic monophyly, phylogeny

## Abstract

The Australian genus *Theprisa* Moore, 1963, is taxonomically revised to comprise five species, two newly described: *Theprisadarlingtoni* Liebherr & Porch, **sp. nov.** of Tasmania, and *Theprisaotway* Liebherr, Porch & Maddison, **sp. nov.** from the Otway Ranges, Victoria. Two previously described species, *T.australis* (Castelnau) and *T.montana* (Castelnau), are distributed in the mountains of Victoria. The third previously described species, *T.convexa* (Sloane) is found in Tasmania. A lectotype is designated for *T.convexa* because the various syntypes are ambiguously labelled. Cladistic analysis based on morphological characters establishes monophyly of *Theprisa* relative to the Australian genera *Sitaphe* Moore and *Spherita* Liebherr. This and a second clade of Australian genera (*Pterogmus* Sloane, *Thayerella* Baehr, and *Neonomius* Moore) do not form a natural group, but are cladistically interdigitated among two monophyletic New Zealand lineages (*Tarastethus* Sharp, and *Trichopsida* Larochelle and Larivière) suggesting substantial trans-Tasman diversification among these groups. Hypothesized relationships within *Theprisa* are consistent with two bouts of speciation involving the Bass Strait; an initial event establishing *T.convexa* as adelphotaxon to the other four species, and a more recent event establishing the sister species *T.darlingtoni* and *T.montana*. Geographic restriction of *T.otway* to the Otway Ranges is paralleled by Otway endemics in several other carabid beetle genera, as well as by endemics in numerous other terrestrial arthropod taxa. Whereas these numerous Otway endemics support the distinctive nature of the Otway Range fauna, their biogeographic relationships are extremely varied, illustrating that the Otways have accrued their distinctive biodiversity via various means.

## Introduction

This revision was initially conceived in a world unlike the one we currently inhabit. As the 2019 coronavirus pandemic spread worldwide, workplaces were shuttered, air travel and transportation changed to essential deliveries, and all of us physically distanced to preserve our collective health. It was also the time that we lost Terry Erwin, taking away a major contributor to the study of biodiversity worldwide (Kavanaugh 2020). A constant theme of Terry Erwin's research was his inevitable response to challenges and adversity. He attacked the challenges directly, taking action to mitigate any negative outcomes, and succeeded by dint of effort and organization. This group of authors took that to heart, and though we are far flung geographically, we assembled a team using the internet to share scientific information, allowing us to remotely assemble the necessary components of this revision. Insect collection curators are charged with both the maintenance and protection of previously described specimens, but also with the development of institutional collections, achieved through use of specimens in support of novel descriptive taxonomy. This activity is increasingly tied to electronic access of collection-based information, yet that information must be of the highest quality to maximally promote truth in science. This contribution brings together information from specimens described during the past 150 years, with those specimens housed in Europe, North America, and Australia. Given the current lockdown of society, only an internet-based effort could produce such a taxonomic product.

The genus *Theprisa* Moore, 1963, was described as a distinct taxon based on characters of the mouthparts and male genitalia (Moore 1963b), yet the taxonomic unit was first recognized informally by Sloane (1920). In a recent cladistic analysis of Moriomorphini Sloane, 1890, the tribe that includes *Theprisa*, monophyly of the genus was proposed but only based on inclusion of two *Theprisa* species (Liebherr 2020). This revision incorporates cladistic analysis including the three previously described species of *Theprisa*, two newly recognized species, as well as representatives of closely related genera. This allows testing monophyly of *Theprisa* and proposing its adelphotaxon. Our geographically diverse team, united by the internet, was able to assemble and share data on all available specimens, including all type specimens held in Australian museums. We dedicate the product of our worldwide collaboration to the memory of Dr. Terry L. Erwin.

## Materials and methods

### Taxonomic material

Prior to the pandemic, JKL had borrowed or examined specimens of *Theprisa* from the following institutional collections: Australian National Insect Collection, Canberra (**ANIC**); Carnegie Museum of Natural History, Pittsburgh, PA (**CMNH**); Cornell University Insect Collection, Ithaca, NY (**CUIC**); Field Museum of Natural History, Chicago, IL (**FMNH**); Museo Civico di Storia Naturale “Giacomo Doria”, Genova, Italy (**MGDG**); Museum of Comparative Zoology, Harvard University, Cambridge, MA (**MCZ**); Zoological Museum, University of Copenhagen Denmark (**ZMUC**).

Museums Victoria, Melbourne (**MVM**) also holds specimens of *Theprisa*, with identities of those specimens assessed by N. Porch using a manuscript version of this contribution. Similarly, four specimens were borrowed by N. Porch from the Tasmanian Museum and Gallery, Hobart (**TMAG**), with their identities determined through shared digital photographs. Specimens collected by N. Porch are deposited in the Nick Porch Collection (**NPC**), Deakin University, Geelong, Victoria, and Museums Victoria. D. R. Maddison studied specimens deposited in the Oregon State Arthropod Collection, Corvallis, OR (**OSAC**) and the Essig Museum of Entomology, University of California, Berkeley (**EMEC**). In sum, the revision was based on examination of 350 *Theprisa* specimens.

Digital photographs of specimens were shared among the authors to confirm species assignments. Authorship of the two new species was conditioned upon authors’ contributions of paratypes to the type series. Additional type material held in **ANIC** was examined by B. Sinclair, and types held in the South Australian Museum, Adelaide (**SAMA**) were examined by M. Shaw. Digital images of all types held in Australian institutions were transmitted to all co-authors allowing consensus on their specific identities (Suppl. material 1). A lectotype of *Phersitaconvexa* Sloane, 1920 is designated, and all paralectotypes are accounted for within the ANIC and SAMA holdings. As these specimens have been variously labelled “type”, “co-type”, “paratype” or “hololecto” by prior workers, the complete label information is provided for each type specimen. Lectotypes were previously designated for names of Castelnau (1867, 1868) by Straneo (1941). All types of previously described taxa have been examined, and their label information is provided below. For each specimen, information on separate labels is separated by a double slash (“//”), and individual lines on a single label are bounded by single slashes (“/”). Holotype and lectotype label data are presented verbatim. Presentation of label data associated with paratypes collected by P. J. Darlington, Jr. is augmented using his field notes (Darlington 1960), with that information presented within brackets.

### Laboratory techniques

Specimens were examined by dissecting microscope, using both fiber-optic dual-wand and ring light sources. Genitalic dissections were undertaken after specimens were relaxed for 2 hrs in near-boiling distilled water including several drops of Kodak Photo-Flo detergent (Rochester, NY). For male specimens the invaginated abdominal segments VIII and IX and the included aedeagus were removed using minuten pins, cleared in cold 10% KOH overnight, neutralized in 10% acetic acid, and examined after 24 hours in glycerin. Female dissections involved removal of the entire abdomen, clearing overnight in cold 10% KOH and neutralization in acetic acid, with isolation of gonocoxae and the associated female reproductive tract and hind gut accomplished using fine forceps and minutens. The reproductive tract was stained for 15–30 mins in Kodak Chlorazol Black mixed into methyl cellosolve, with the stained preparation placed into glycerine for 24 hours prior to continued dissection.

Photographs of whole specimens and dissected genitalia and reproductive tracts were made using a Microptics macrophotographic apparatus, mounting a Nikon D1 camera and Microptics K2 lens system, with lighting provided by a three wand fiber-optic photographic strobe unit allowing either direct lighting or transmitted light on a microscope slide stage. Images were captured and stacked using Compose Z5 software (Hadley 2006).

### Morphological characters

For diagnostic purposes, various ratios of body dimensions are useful, including those of the head, pronotum, and elytra. Eye development is characterized by the ocular ratio, or maximal head width across the compound eyes divided by the minimal frons width between the eyes. The eyes are borne on an ocular lobe, with the amount of that lobe covered by the eyes quantified as the ocular lobe ratio (**EyL/OLL**), or the length of the eye measured in dorsal view divided by the distance from the front of the eye to the juncture of the ocular lobe and gena behind the eye. Eye convexity is quantified as the length of the eye in dorsal view divided by depth of the eye (**EyL/EyD**), that depth measured with the eye's, dorsal margin oriented uppermost in the field of view. Pronotal shape can be used to diagnose species, with **MPW/PL**, or maximal pronotal width divided by median pronotal length assessing the relative length of the pronotum, and **MPW/BPW**, or maximal pronotal width divided by basal pronotal width between the hind angles assessing the basal constriction of the pronotum. Elytral configuration is assessed using **HuW/MEW**, or the distance between the humeral angles divided by maximal elytral width, and **MEW/EL**, or maximal elytral width divided by elytral length, or the distance from the basal declivity of the scutellum to the apex of the longer elytron just laterad the suture. Standardized body length is quantified as the sum of the distance from the anterior margin of the labrum to the cervical ridge at the back of the vertex, plus **PL** and **EL** defined above. For all measurements, the number of individuals measured to estimate variation is indicated parenthetically at the start of the diagnosis.

Cuticular microsculpture can be used to assist diagnosis, with terms for the shapes of sculpticells following Lindroth (1974) and Allen and Ball (1980). Male genitalic armature includes a flagellum on the internal sac (Maddison 1993), with the configuration of that structure providing diagnostic information in both the uneverted and everted condition. Presentation of female gonocoxal setation follows Ball and Shpeley (1983), and female reproductive tract characters, Liebherr and Will (1998). The numbers of individuals dissected to determine genitalic characters is indicated parenthetically at the start of each male genitalia and female reproductive tract section of the descriptions.

Full descriptions of both external and internal genitalic and productive tract characters are presented for both new species. For the previously described species, an extended diagnosis based on external characters is complemented by a full description of the genitalic and reproductive tract characters.

### Phylogenetic analysis

Liebherr (2020) presented a morphologically based cladistic analysis of the Moriomorphini, establishing monophyly for genera comprising the tribe. *Theprisa* was placed in that analysis as a member genus of subtribe Tropopterina, with the two generic representative species placed along a stem of the tree amongst representatives of other genera from Australian and New Zealand (Liebherr 2020: fig. 1). Liebherr (2019), based on personal communication from D. R. Maddison, assessed the inclusion of *Thayerella*Baehr (2016) in Moriomorphini, concluding that this taxon is a member of a clade also including *Pterogmus* and *Neonomius*. Given that the goal of this contribution was revision of *Theprisa*, the question of *Theprisa* monophyly gains importance as this paper recognizes five species, two new, to expand the representation of species in Liebherr (2020). As such, phylogenetic analysis focused on *Theprisa* and its cladistic neighbors is presented to allow assessment of generic monophyly based on the five currently recognized species of *Theprisa*. The 16 taxa chosen to accompany the species of *Theprisa* in this analysis included representatives of the following genera: *Molopsida* White, *Tarastethus* Sharp, *Trichopsida* Larochelle and Larivière, *Pterogmus* Sloane, *Neonomius* Moore, *Spherita* Liebherr, and *Sitaphe* Moore. These genera bracket *Theprisa* by two cladogram nodes in either direction from the attachment point of *Theprisa* to the Hennigian comb defining mid-grade members of the subtribe Tropopterina (Liebherr 2020: fig. 1). Taxa sampled from these genera were also restricted to those represented by both male and female specimens. Removing these taxa represented partially by missing data eliminated topological ambiguity from the analysis. By this taxonomic sampling, the goal of testing *Theprisa* monophyly was undertaken within a reasonable sampling of putatively related taxa, based on complete morphological data for the representative taxa.

The present cladistic analysis is based on 110 characters; 105 ordered binary or multistate, and five unordered multistate characters. The matrix was constructed by culling taxa and associated uninformative characters from the data matrix of Liebherr (2020), while adding character information that includes several relevant new characters for the three additional *Theprisa* spp. plus *Thayerellanewtoni* Baehr. Character information was managed using Winclada (Nixon 2002), with analyses run using NONA (Goloboff 1999) and the parsimony ratchet (Nixon 1999) undergoing 200, 1000, and 10,000 iterations. All character and taxon data can be viewed in Suppl. material 2; i.e., the Winclada file with resultant trees derived from the 10,000-iteration ratchet run. Though identical results were obtained under all numbers of iterations of the ratchet, the parsimony results were checked and found identical using TNT (Goloboff et al. 2008; Goloboff and Catalano 2016), running a new technology sectorial search, with ratchet, drift, and tree fusing options; initial addseqs. = 10 and random seed = 1. The matrix was also analyzed with PAUP (Swofford 2002), finding the identical set of parsimonious trees, while also generating decay indices (Bremer 1994) for all internal branches of the cladogram.

## Taxonomic treatment

### Theprisa

Taxon classificationAnimaliaColeopteraCarabidae

Moore, 1963

5E538809-D5A0-54AE-A5E1-699E3DA6C600

Theprisa Moore, 1963b: 285 (type species Phersitaconvexa Sloane, 1920: 156) by original designation.

#### Diagnosis.

This genus is diagnosable within Moriomorphini by: 1, mesosternum broad between mesocoxae; 2, mandibular scrobe present, margined ventrally by a lateral expansion; 3, mesotibia gracile, not expanded apically; 4, apical two maxillary palpomeres and apical labial palpomere apparently glabrous; 5, prosternal process not margined apicoventrally; 6, elytral striation nearly complete, striae 1–7 all developed apically, and striae 6–8 as deep or only slightly shallower than striae 1–5 near elytral midlength (Liebherr 2020). The mandibles are moderately elongate, with the mandibular length measured from the anterior condyle 1.7–1.9× distance from anterior condyle to apicolateral margin of labrum. The ligula is broadly rounded to truncate apically, bisetose, with the two setae separated by 2–3 diameters of the setal articulatory sockets. The paraglossae are elongate and thin, their overall length 2–3× the distance from the paraglossal base to the apical ligular margin. The mentum tooth is present, its apex narrowly rounded to subacuminate and its lateral margins upraised. Both lateral and basal pronotal setae are present. The elytra lack dorsal setae except for *T.otway* sp. nov., which has a single dorsal seta near midlength. The parascutellar seta is present. There are 13 lateral elytral setae associated with the eighth stria, arranged in an anterior series of seven setae and a posterior series of six, and both subapical and apical elytral setae are present. As in many moriomorphines, the apical portion of interval 8 immediately laterad stria 7 is upraised as a distinct carina. Also, the metathoracic flight wings are vestigial in all *Theprisa*, with the metepisternum foreshortened; its maximum width subequal to greater than the lateral length.

### Key to adults of *Theprisa* Moore

**Table:** 

1	Body more gracile, MPW/PL = 1.25–1.34; elytra ovoid to subquadrate, third interval glabrous (Figs 1B, C, 2); prosternum impunctate across anterior surface	**2**
–	Body broad, MPW/PL = 1.38–1.46; elytra broadly hemi-ovoid, a single seta present mesad third stria at 1/3 elytral length (Fig. 1A); prosternum irregularly depressed each side anterad coxal cavity, ~8 punctures associated with transverse to oblique depression; male aedeagal median lobe with broad invagination, or divot, along ventral portion of apical margin (Fig. 3A)	***Theprisaotway* sp. nov.**
2	Elytral striae smoother, wavering slightly to straight in deepest portions (Figs 1C, 2); elytral lateral margins subparallel to moderately convex at midlength, HuW/MEW = 0.64–0.67; pronotal basal margin straight to trisinuate, margin behind laterobasal depressions not distinctly angled anteriorly relative to median base; aedeagal median lobe apex blunt apically, not evenly rounded (Fig. 3C–E)	**3**
–	Elytra striae distinctly crenulate, punctures visibly expanding breadth (Fig. 1B); elytral ovoid, base constricted, humeri narrow, HuW/MEW = 0.60–0.61; pronotal basal margin distinctly angled laterad median base and slightly concave posterad laterobasal depressions, thus median base extended posteriorly; aedeagal median lobe apex broadly, evenly rounded (Fig. 3B)	***Theprisaconvexa* (Sloane)**
3	Pronotal lateral margin straight or nearly straight anterad hind angle, curvature of margin associated only with projection of the basal seta articulatory socket and not extended beyond that projection (Fig. 2); aedeagal median lobe flattened apically, apical face present (Fig. 3D, E)	**4**
–	Pronotal lateral margin distinctly sinuate anterad hind angle, sinuation extended well forward of hind seta articulatory socket (Fig. 1C); aedeagal median lobe apex narrowly rounded (Fig. 3C)	***Theprisamontana* (Castelnau)**
4	Pronotal base broadly punctate both on median base and laterally, laterobasal depression continuous from median base to near hind angle, with only a low tubercle laterad mesal longitudinal depression (Fig. 2A); standardized body length smaller, 5.7–7.1 mm; male aedeagal median lobe apex broad with a straight apical face, broad surfaces of lobe apex bearing large pitted depressions (Fig. 3D)	***Theprisaaustralis* (Castelnau)**
–	Pronotal base smooth to minutely punctate, laterobasal depression composed of deep inner longitudinal depression bordered laterally by a well-developed tubercle that is continuous with pronotal disc (Fig. 2B); body size larger, standardized body length 7.4–8.0 mm; male aedeagal median lobe apex with thickened ventral margin (Fig. 3E), tip appearing mucronate	***Theprisadarlingtoni* sp. nov.**

### Theprisa otway

Taxon classificationAnimaliaColeopteraCarabidae

Liebherr, Porch & Maddison sp. nov.

936E7F35-4643-54FD-A3AA-E17365619711

http://zoobank.org/5B055C0A-CC24-44E3-B32B-2D04785D52A7

Figures 1A , 3A , 4A , 5A , 6A , 7A , 8A , 9

#### Holotype

♂ (ANIC). 38.39S 143.42E VIC / Haines Junct. 525 m 1.9 km / W. on Turton's, Track / 809 26Jan.-8Feb. 1987 / A. Newton & M. Thayer // wet scler. forest / pyrethrin fogging / fungus logs / Euc. regnans // Theprisa otway sp. n. / measured specimen #1 / det. J.K. Liebherr 2020 // HOLOTYPE ♂ / Theprisa otway / J.K. Liebherr, N. Porch / D.R. Maddison 2020 (black-margined red label).

#### Paratypes.

Victoria: Otway Ranges [sclerophyll forest approaching rain forest in ravines, some *Nothofagus*; logs, stones, drowning; ~ 600 m (Darlington 1960)], Darlingtons [12-13]-iv-1957 (MCZ, 11); Otway N. P., Elliot R. 5.5 km W Marengo, wet sclero. forest, FMHD #87-262, Berl. leaf and log litter, 38°47'S, 143°37'E, 80 m, Newton & Thayer, 8-ii-1987 (FMNH, 3), roadside, C119 1 km S Tanybryn, road to Sabine Falls, pyr. fogging mossy logs, 38°37.07'S, 143°43.80'E, Seago & Brandley, 28-xi-2006 (EMEC, 1; OSAC, 1), Triplet Falls track, *Eucalyptus* forest, raking litter, 38°40.235'S, 143°29.690'E, 300 m, Liebherr, 14-ii-2011 lot 03 (CUIC, 1), Turton's, track, 1 km ENE, Seaview Ridge Road junction, Mountain Ash forest, berlesate of deep litter against tree and under treeferns, 38°38.34'S, 143°37.30'E, 505 m, Porch, 15-iii-2019 (MVM, 1; NPC, 1).

#### Diagnosis

**Figure 1.**
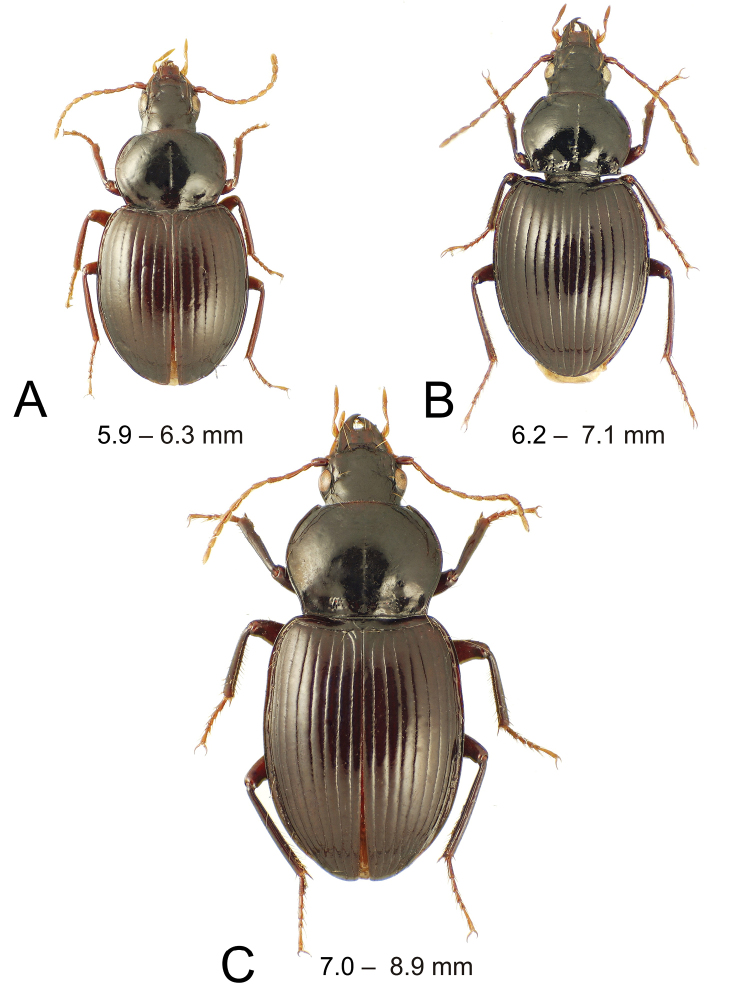
Dorsal habitus illustrations of *Theprisa* spp.; range of standardized body lengths indicated **A***T.otway* female **B***T.convexa* female **C***T.montana* female.

(n = 5). This species is diagnosable among *Theprisa* spp. by the broad body and the presence of a dorsal elytral seta immediately mesad the third stria near elytral midlength (Fig. 1A). The pronotum is broad, MPW/PL = 1.38–1.46. The pronotal median base is sparsely punctate medially, with the marginal bead greatly reduced, evidenced only as a broad, slightly depressed area along the medially expanded base, in contrast to the narrowly upraised, distinct marginal bead posterad the laterobasal depressions. Whereas elytral striae 1–4 are crenulate on the disc (i.e., lined by punctures that expand the strial breadth) striae 5–7 are progressively broader and smoother, 6–7 slightly wavering along length but impunctate. In contrast *T.australis*, *T.darlingtoni*, and *T.montana* have striae 1–7 smooth, and *T.convexa* has striae 1–7 punctate near midlength. Apical abdominal ventrite of male with a single seta each side along margin, female apical ventrite with two setae each side plus a median pair of subapical setae. Standardized body length is 5.9–6.3 mm.

#### Description.

***Head*** narrow, ocular lobe little projected, juncture with gena very obtuse; eyes small, EyL/OLL = 0.65–0.70, not projected beyond curvature of ocular lobe, 17–20 ommatidia bisected by a horizontal line across eye; antennomeres 2 and 3 glabrous except for one dorsal seta on 2 and an apical ring of setae on 3; antennae moderately elongate, antennomere 9 maximal breadth 2× length; frontal groove deeply, medially arcuate from anterior supraorbital seta to frontoclypeal suture and continued onto lateral reaches of clypeus, area laterad groove distinctly convex; slightly concave apical labral margin 6-setose, with three smaller setae lining the lateroapical margin; mentum tooth narrowly rounded apically, sides subparallel; maxillary stipes trisetose, the three setae on the base in either a triangle with apex upward, or in an irregular horizontal line; ligula slightly convex apically, narrowed basally, trumpet shaped, its two apical setae separated by three setal diameters; paraglossae elongate, total length 2.5× distance from paraglossal base to ligular apical margin. ***Pronotum*** transverse, lateral margins straight anterad projection defined by articulatory socket of basal seta; basal margin nearly straight, the obsoletely margined median base projected posterad only slightly beyond the lateral beaded margins posterad the laterobasal depressions; median base smooth at middle, ~ 10 small punctures each side mesad deepest point of laterobasal depression; pronotal disc extended to basal marginal bead defining a tubercle that divides laterobasal depression into a median longitudinal groove and a broad lateral marginal depression inside the basal seta; median longitudinal depression deepest just anterad median base, deeply incised to very shallow, broad anterior transverse impression; anterior callosity nearly flat, front margin smooth medially, margined in outer half each side; front angles broadly, slightly protruded, subangulate; lateral marginal depression very narrow from front angle to basal 1/4 of length where it expands to meet laterobasal depression; lateral pronotal seta positioned one setal diameter inside lateral marginal depression. ***Prosternum*** medially depressed mesad anterior margins of procoxal cavities, up to 16 punctures present in a transverse band across apical 1/4 of prosternum, ~ 10 punctures each side of prosternum in depressed area anterad coxal cavity; proepisternum smooth, sutural groove with proepimeron smooth and deep. ***Elytra*** broadly hemi-ovoid, MEW/EL = 0.81–0.86, convex, sides vertically meeting lateral marginal depression; basal groove arcuate, juncture with lateral marginal depression tightly rounded, a broad, blunt tooth on margin at juncture; lateral marginal depression narrow; stria 8 deep, continuous between anterior and posterior series of lateral setae; apical carina of interval 8 narrowly upraised along stria 7, interval 8 a vertical lateral carina there; subapical sinuation evident, the internal plica visible ventrad deepest part of sinuation. ***Mesepisternum*** with ~ 8 shallow punctures in two vertical rows. ***Metepisternum*** wider than long, maximum width 1.8× lateral length; metepimeron fused to metepisternum laterally. ***Legs*** gracile, femora narrow, meso- and metatibiae little expanded apically, of consistent diameter throughout apical half of length; metatarsomere 1 elongate, length 0.22× tibial length, lateral sulci present on mesal and lateral faces just dorsad the ventrolateral setae. ***Abdominal ventrites*** 2–6 with 1–2 longitudinal wrinkles laterally; suture between ventrites 1 and 2 slightly curved anteriorly at midlength, ventrite 2 wrinkled within curve; suture between ventrites 2 and 3 complete laterally.

***Male genitalia*** (n = 2). Aedeagal median lobe robust, base broadly open on right side, basal margin thickened dorsad basal opening (Fig. 3A); median lobe apex broadly rounded, slightly extended beyond ostium with a large apical divot-like concavity at tip, surface of apex densely covered with pits; internal sac bearing a stout flagellum, with apex concavely scooped (Fig. 4A), a basal articulatory sclerite associated with flagellum; right paramere elongate, parallel sided in apical half with apex narrowly rounded (Fig. 5A), bearing 12–15 short setae along ventral margin in apical half, 2–4 setae on dorsal surface near apex, though with apex glabrous; left paramere broadest near midlength, apex narrower, parallel sided with rounded tip, ventral margin bearing 2–4 setae in apical 1/3, dorsal surface with two or three setae near apex, and apex with none or one seta; antecostal apodeme of abdominal segment IX rounded distally, the apical juncture of lateral arms broad (Fig. 6A).

***Female reproductive tract*** (n = 2). Bursa copulatrix columnar, length 1.25× maximum breadth compressed under microslide cover slip, vagina translucent, broader apical portion of bursa staining more darkly with Chlorazol black (Fig. 7A); helminthoid sclerite present, rounded apically, not extended beyond juncture with spermathecal duct; spermathecal duct stout, sinuously recurved to meet spermatheca, length subequal to length of annulated spermathecal reservoir; spermathecal gland duct very thin, length twice that of spermathecal reservoir which it joins at reservoir base; spermathecal gland comprising sclerotized stem plus membranous reservoir bearing numerous ductules; gonocoxa bipartite, basal gonocoxite 1 with two apical fringe setae in one dissected specimen, two setae on right gonocoxa and one on left in second dissected specimen, median surface glabrous, membranous ramus present (Fig. 8A); apical gonocoxite 2 with base extended laterally, lateral margin arcuate, apex falciform; two lateral ensiform setae and one dorsal ensiform present; two apical nematiform setae set in fossa at apical 1/4 of apical gonocoxite length.

**Figure 2.**
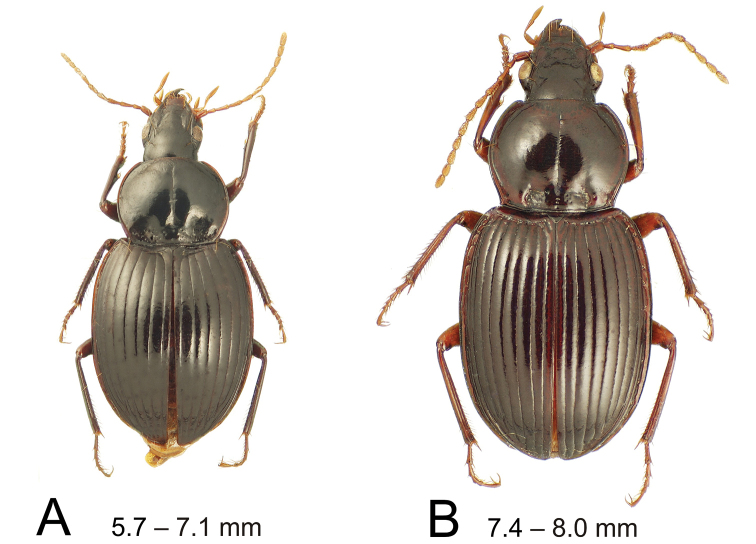
Dorsal habitus illustrations of *Theprisa* spp.; range of standardized body lengths indicated **A***T.australis* male **B***T.darlingtoni*, female.

#### Etymology.

The species epithet is the mountain range from which this species is described, and based on current knowledge, precinctive. The epithet is to be treated as a noun in apposition.

#### Distribution and habitat.

*Theprisaotway* is restricted to the Otway Ranges (Fig. 9), with specimens collected in wet to mesic sclerophyll forest from 80–525 m elevations. The beetles are terrestrial, being discovered via raking litter, in a Berlese extraction of leaf and log litter, and via application of pyrethrin fog to logs of *Eucalyptusregnans* F. Mueller covered with fungus. The MCZ specimens were collected by the Darlington family in sclerophyll forest approaching rain forest in ravines, with some *Nothofagus* (Darlington 1960).

### Theprisa convexa

Taxon classificationAnimaliaColeopteraCarabidae

(Sloane)

472CE671-4639-5350-A146-D7C06A1AB419

Figures 1B , 3B , 4B , 5B , 6B , 7B , 8B , 9

Phersita convexa Sloane, 1920: 158.
Theprisa convexa Moore, 1963b: 285.

#### Types.

***Lectotype*** male (SAMA) labelled by P. J. Darlington, Jr. hereby designated: card-mounted specimen with right antenna broken off distad pedicel, left mesoleg absent // Type // T. / Zeehan // Phersita / convexa Sl. / Id. by T. G. Sloane // Lectoholo. / P. convexa / PJD Sl. [red paper label] // Phersita 9.11564 / convexa Sl. / Tasmania / TYPE [red ink vertically at right end of label] // SAMA Database No. 25-035536 // LECTOTYPE ♂ / Phersita / convexa / Sloane / M. Shaw & J. K. Liebherr 2020 [black-margined red label]. By this action Zeehan, Tasmania is designated type locality.

Sloane stated that this species was described from specimens inhabiting “Zeehan (Simson, No. 2123); Strahan and Waratah (Carter and Lea). Eleven specimens have been examined. (Sloane 1920: 158).” We account below for the 10 paralectotypes to accompany the lectotype in the type series to ensure that this species did not include a specimen of the Tasmanian *T.darlingtoni*, sp. nov., described below. Paralectotypes are numbered to correspond with a gallery of images of the specimens and labels (Suppl. material 1). The first three paralectotypes are deposited in the Sloane Collection (ANIC): **1** and **2**, male (left) and female (right) mounted on single card] // Strahan / Tas : Lea / & Carter // Phersita / convexa Sl. / Id. by T. G. Sloane. // Co-type // ***Paratype*** [blue label of no significance as specimen is part of syntype series] // red rectangle with three holes // ***Paralectotype*** ♀, ♂ / Phersita / convexa Sloane / B. Sinclair & J. K. / Liebherr 2020 [black-bordered red label]; **3**, female: Zeehan / 12/91 [on reverse] // Phersita / convexa Sl. / Id. by T. G. Sloane. // Co-type // ***Paratype*** // ***Paralectotype*** ♀ [as above]. The other seven paralectotypes that accompany the lectotype (SAMA) include: **4**, male card mounted // Zeehan // Phersita / convexa Sl. / Id. by T. G. Sloane // SAMA Database / No. 25-039812; **5** and **6**, two teneral females separately card mounted, on single pin // 3123 [blue paper label] // Tasmania / A. Simson (2 labels) // Phersita / convexa Sl. / Id. by T. G. Sloane // SAMA Database / No. 25-039813 // SAMA Database / No. 25-039814; **7** and **8**, two specimens, gender not determined, separately card mounted, on single pin // Tasmania / A. Simson (2 labels) // 3123 [blue paper label] // Phersita 19594 / convexa Sl. / Id. by T. G. Sloane / Cotype [red ink vertically on right end of label] // SAMA Database / No. 25-039819 // SAMA Database / No. 25-039820; **9** and **10**, two specimens mounted on one card, gender not determined // Waratah / Tas: Lea / & Carter (2 labels) // Phersita / convexa Sl. / Id. by T. G. Sloane // Co-type // // SAMA Database / No. 25-039821 // SAMA Database / No. 25-039822. All paralectotypes in SAMA also bear a bottom label, one per pin: ***Paralectotype* (*s*)** / Phersita / convexa Sloane / M. Shaw & J. K. Liebherr 2020 [black margined red label]. The discrepancy in recorded lot numbers 2123 in the published description versus 3123 on the specimen labels is adjudicated in favor of the specimen labels. Looking through registers at SAMA, we have not been able to locate this number, nor several similar missing numbers that also relate to various Tasmanian beetles from Simson.

#### Extended diagnosis

(n = 5). This species is aptly named due to the convex, domed elytra with depressed scutellar area (Fig. 1B). The elytra are narrowed basally, with the angulate humeri constricted laterally; HuW/MEW = 0.61. In keeping with the basally constricted elytra, the pronotum is more cordate than in the other species (MPW/BPW = 1.23–1.31) and the lateral margins are sinuate anterad the pronotal basal seta articulatory socket. The pronotal base is also unique in the genus, with the marginal bead continuously marked across its breadth, and the lateral portions of the basal margin posterad the laterobasal depressions angled anteriorly relative to the extended median base. The laterobasal depression is broadly quadrate, with a moderately upraised, punctate tubercle bordered medially by a deep impression laterad the median base, and laterally by a narrow longitudinal depression inside the broadly elevated lateral margin. The body surface is glossy, with the vertex covered with indistinct transverse lines joined into an elongate transverse mesh in parts, the pronotal disc glossy with micropunctures visible across the disc, and the elytral disc and apex subiridescent due to indistinct transverse line microsculpture. Apical abdominal ventrite of male with two setae each side along margin plus two widely spaced medial subapical setae nearly in line with the outer four apical setae, female apical ventrite with two setae each side plus a transverse line of three or four medial subapical setae. Standardized body length is 6.2–7.2 mm.

**Figure 3.**
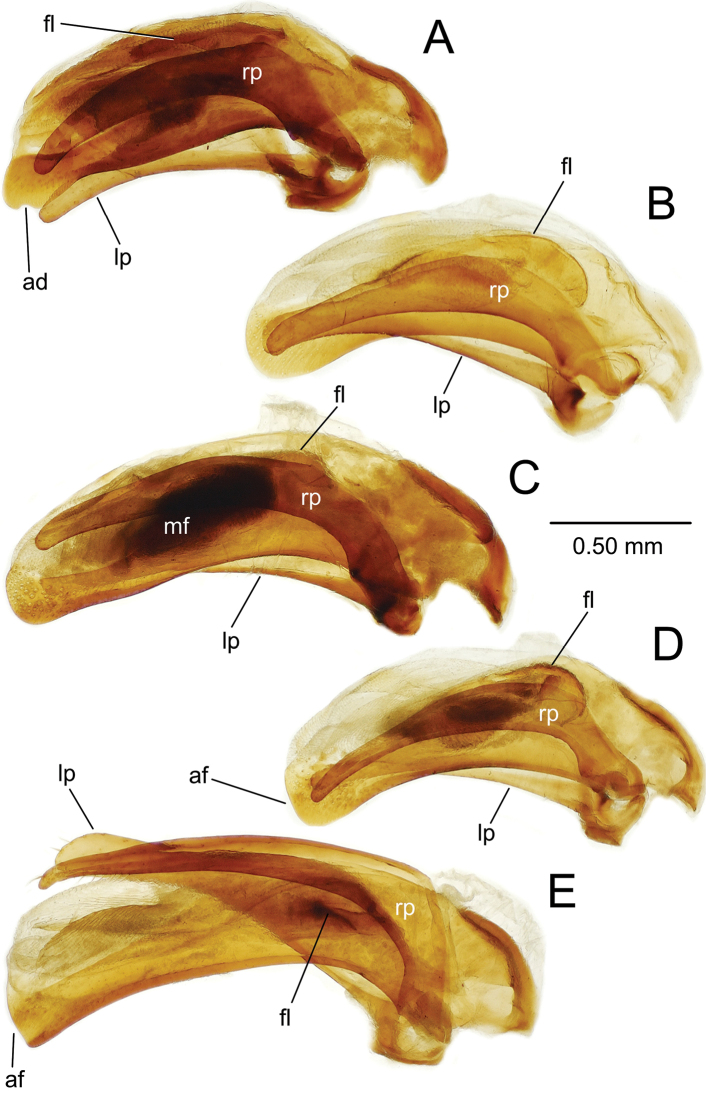
Male aedeagus including parameres of *Theprisa* spp., right, or anatomically ventral view **A***T.otway***B***T.convexa***C***T.montana***D***T.australis***E***T.darlingtoni*. Abbreviations: ad, apical divot; af, apical face; fl, flagellum; lp, left paramere; mf, microtrichial field; rp, right paramere.

**Figure 4.**
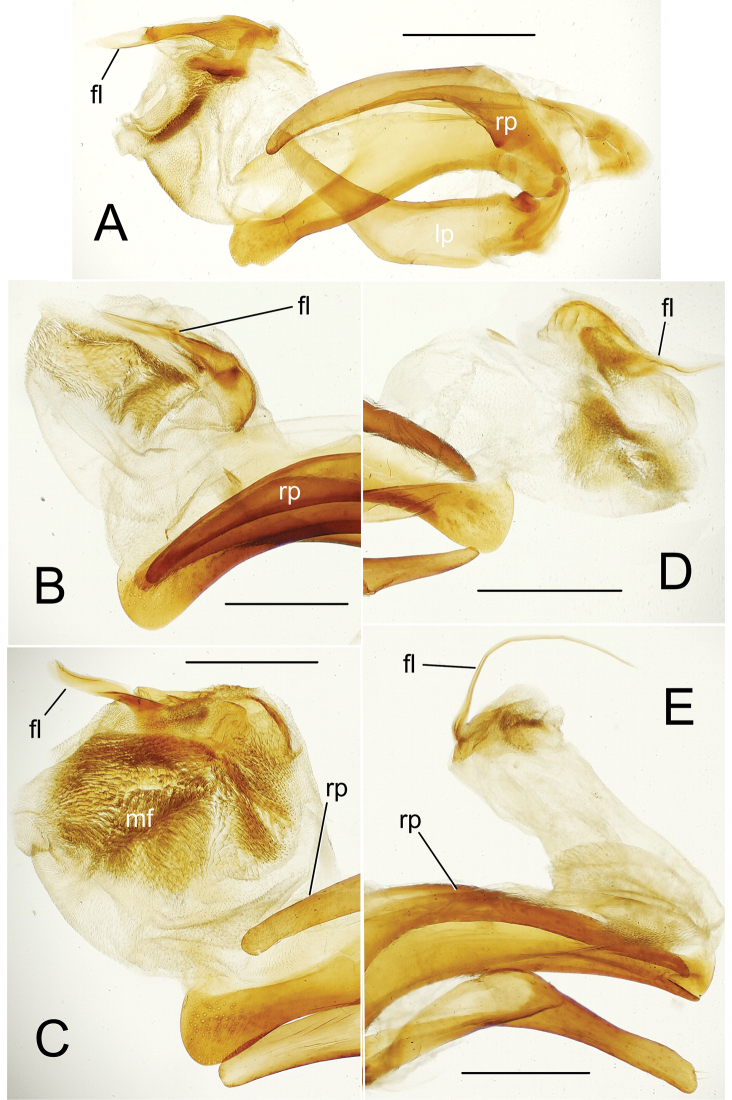
Male aedeagus internal sac in everted condition for *Theprisa* spp. **A***T.otway*, right view **B***T.convexa***C***T.montana***D***T.australis***E***T.darlingtoni*. Abbreviations: fl, flagellum; mf, microtrichial field; rp, right paramere. Scale bars: 0.5 mm

**Figure 5.**
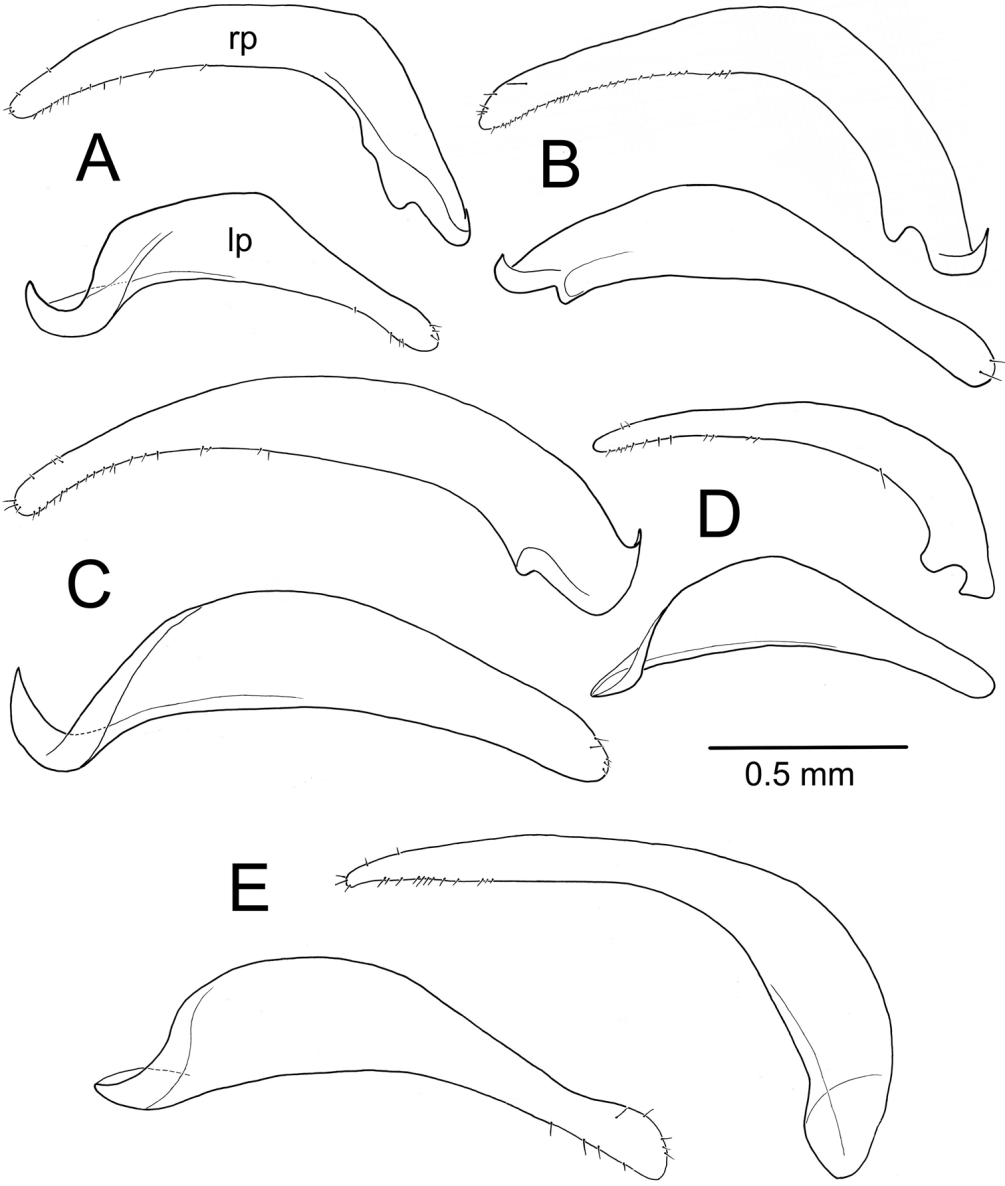
Parameres of male aedeagus for *Theprisa* spp., outside lateral view. Right paramere shown above left paramere **A***T.otway***B***T.convexa***C***T.montana***D***T.australis***E***T.darlingtoni*. Abbreviations: lp, left paramere; rp, right paramere.

**Figure 6.**
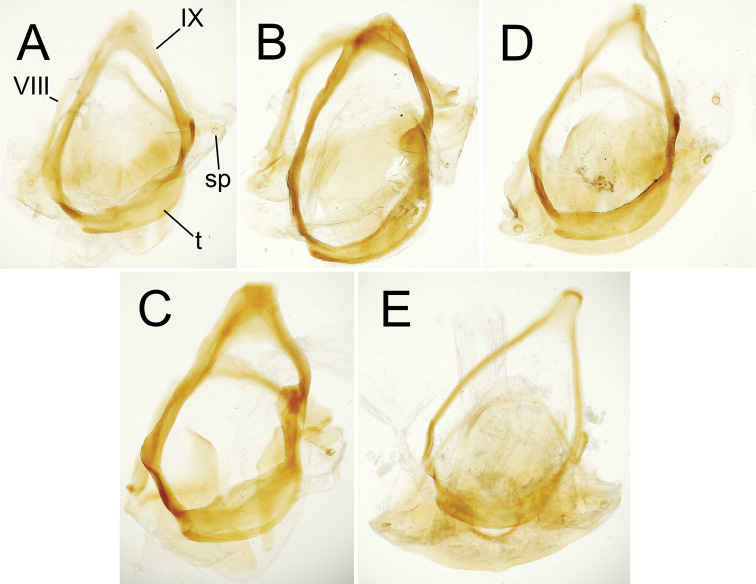
Structures associated with abdominal segments VIII and IX for *Theprisa* spp., dorsal view **A***T.otway***B***T.convexa***C***T.montana***D***T.australis***E***T.darlingtoni*. Abbreviations: VIII, antecostal apodeme of abdominal segment VIII; IX, antecostal apodeme of abdominal segment IX; sp, spiracle of segment VIII; t, tergite of segment IX. Scale bars: 0.5 mm.

***Male genitalia*** (n = 6). Aedeagal median lobe robust, base broadly open on right side, basal margin extended as a sagittal crest dorsad basal opening (Fig. 3B); median lobe apex broadly and evenly rounded, extended only slightly beyond ostium, lateral surfaces of apex densely covered with large pits; internal sac bearing an apically narrowed lanceolate flagellum (Fig. 4B), its broadly sclerotized base extremely evident in uneverted specimens (Fig. 3B); right paramere elongate, evenly curved along length, gradually narrowed in apical half to tightly rounded tip (Fig. 5B), bearing 19–29 short setae along ventral margin in apical half, 2–9 setae on dorsal surface near apex, and with apex bearing 0–2 setae; left paramere slightly broader near midlength, apex slightly constricted before rounded apex, ventral margin usually glabrous (one individual with a single short ventral seta, a second with nine short setae), dorsal surface with 0–5 setae near apex, and apex with 0–2 setae present; antecostal apodeme of abdominal segment IX rounded distally, the apical juncture of lateral arms broad (Fig. 6B).

**Figure 7.**
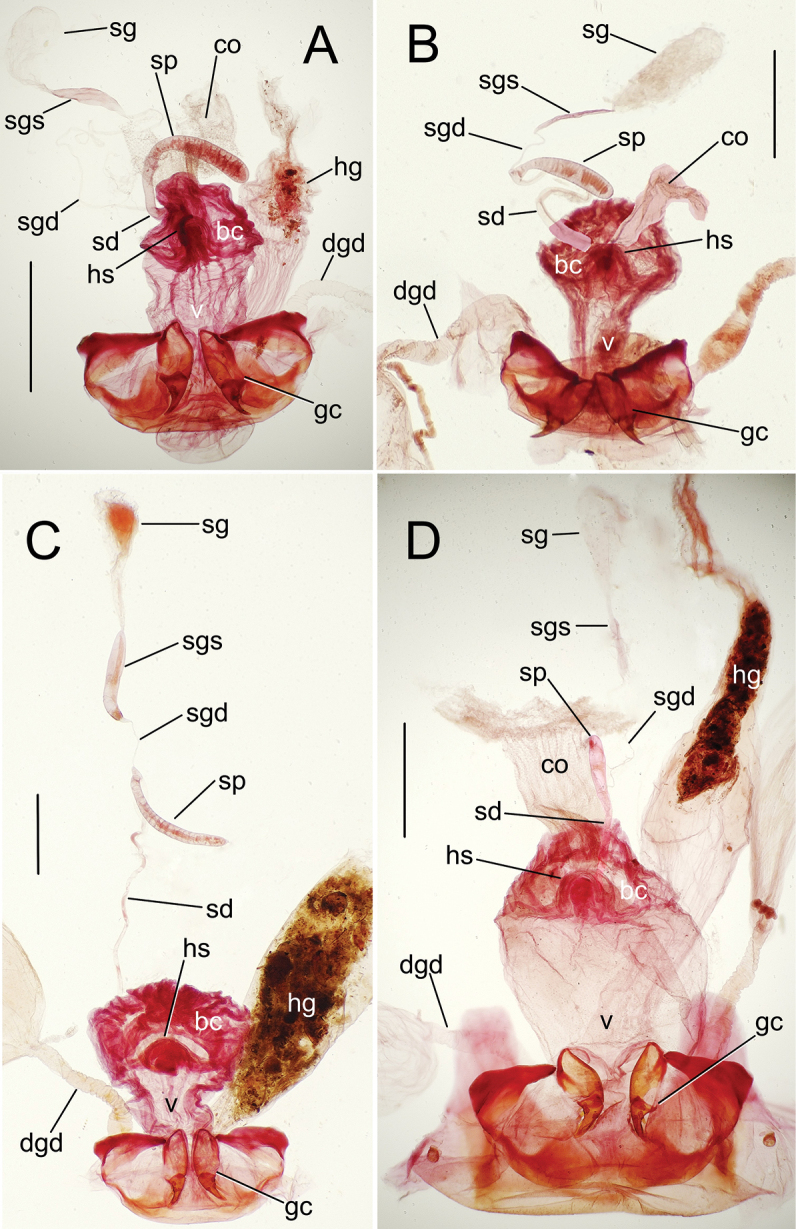
Female reproductive tract of *Theprisa* spp., ventral view **A***T.otway***B***T.convexa***C***T.montana***D***T.australis*. Abbreviations: bc, bursa copulatrix; co, common oviduct; dgd, defensive gland efferent duct; gc, gonocoxa; hg, hindgut; hs, helminthoid sclerite; sd, spermathecal duct; sg, spermathecal gland; sgd, spermathecal gland duct; sgs, spermathecal gland stem; sp, spermatheca; v, vagina. Scale bars: 0.50 mm.

**Figure 8.**
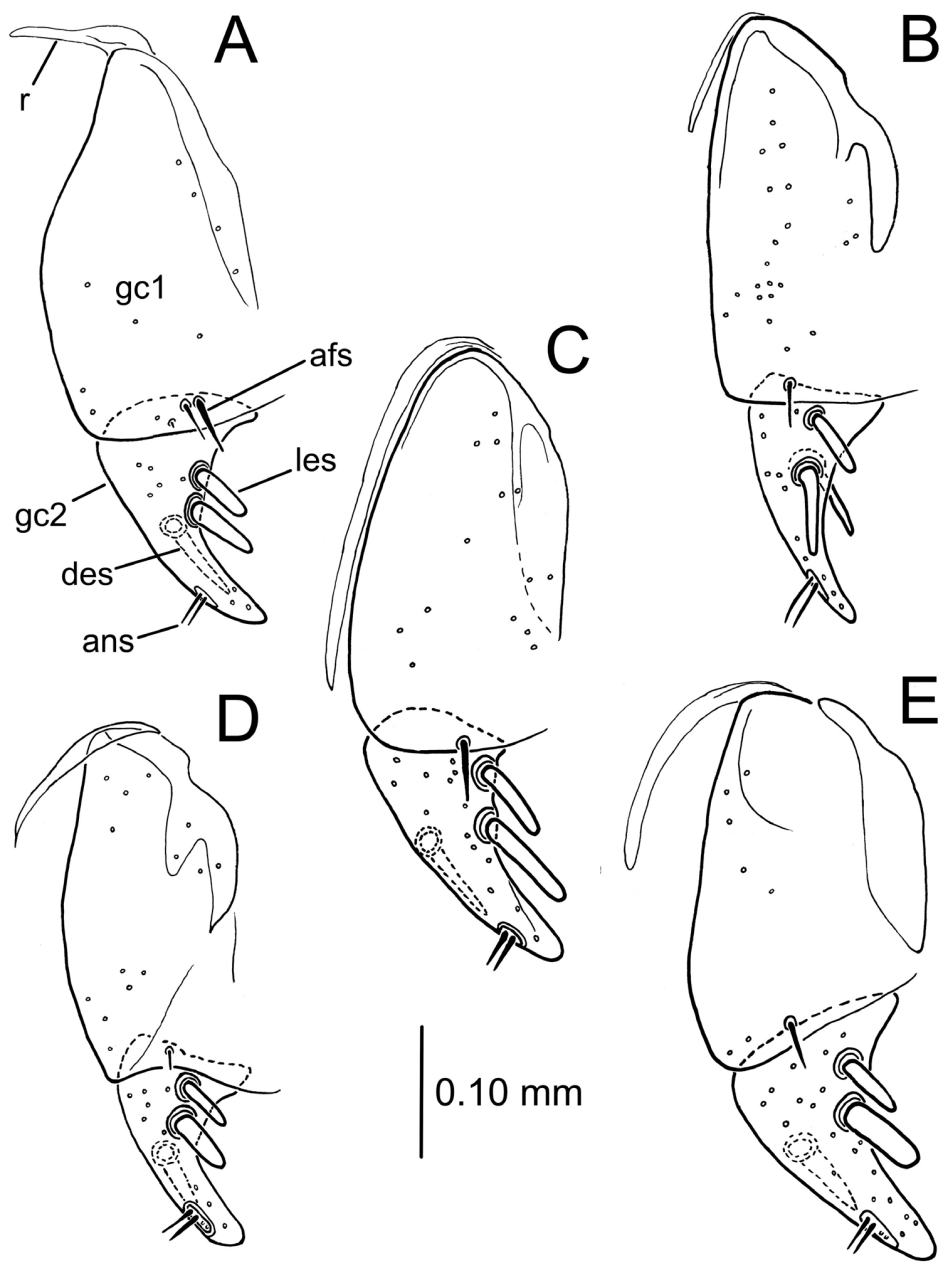
Left gonocoxa, ventral view of *Theprisa* spp., ventral view **A***T.otway***B***T.convexa***C***T.montana***D***T.australis***E***T.darlingtoni*. Abbreviations: afs, apical fringe seta(e) of gonocoxite 1; ans, apical nematiform setae; des, dorsal ensiform seta; gc1, basal gonocoxite 1; gc2, apical gonocoxite 2; les, lateral ensiform setae; r, ramus.

***Female reproductive tract*** (n = 1). Bursa copulatrix of vase-like configuration, vaginal area constricted relative to broader distal portion of bursa, length 1.25× maximum breadth compressed under microslide (Fig. 7B); helminthoid sclerite present, rounded apically, not extended beyond juncture with spermathecal duct; spermathecal duct stout, sinuously recurved to meet spermatheca, length twice that of annulated spermathecal reservoir; spermathecal gland duct very thin, length half that of spermathecal reservoir which it joins at reservoir base; spermathecal gland comprising sclerotized stem plus membranous reservoir bearing numerous ductules; gonocoxa bipartite, basal gonocoxite 1 with single apical fringe seta, median surface glabrous, membranous ramus present (Fig. 8B); apical gonocoxite 2 with base narrow, lateral margin arcuate, apex acuminate; two narrow lateral ensiform setae and one dorsal ensiform present; two apical nematiform setae set in fossa at apical 1/4 of apical gonocoxite length.

#### Distribution and habitat.

**Figure 9.**
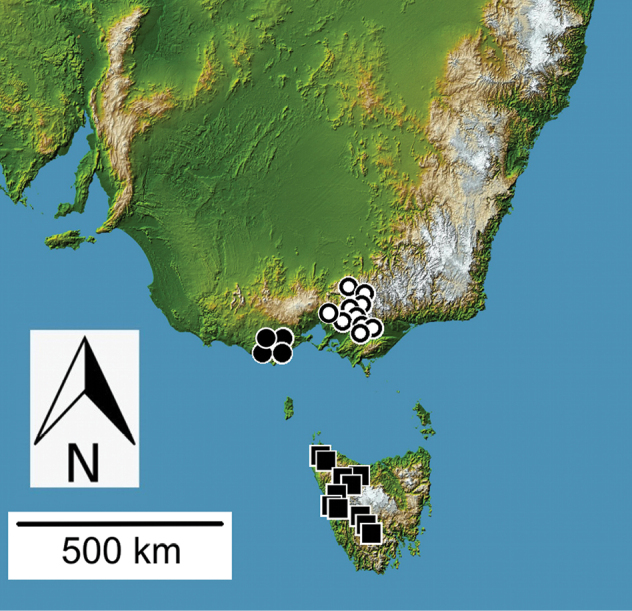
Distributional records for *Theprisa* spp.: *T.otway* (black circle); *T.convexa* (black square); *T.montana* (white circle).

*Theprisaconvexa* is known from the mountainous western portions of Tasmania (Fig. 9), within the West, King, Central Highlands, and Northern Slopes biogeographic regions (IBRA 2012). The beetles are terrestrial, with specimens recorded from under decaying logs, in leaf litter associated with *Nothofaguscunninghamii* (Hooker) (Nothofagaceae), and from riverine forest with *Eucryphia* (Cunoniaceae), *Richea* (Ericaceae), *N.cunninghamii*, and tree ferns. Recorded habitats range 80–1200 m elevation. We have not had the opportunity to examine specimens reported by Eberhard and Giachino (2011), however the geographical expanse of collecting sites they reported agrees totally with our data, with the exception of additional outlying records herein from Christmas Hills, 35 km SW Smithton (Kethley, FMNH), and Trowutta (Madden, TMAG), northwest Tasmania (Fig. 9).

### Theprisa montana

Taxon classificationAnimaliaColeopteraCarabidae

(Castelnau)

FAC55FB0-5711-5652-9595-116DA0E43957

Figures 1C , 3C , 4C , 5C , 6C , 7C , 8C , 9

Drimostoma montana Castelnau, 1867: 112.
Drimostoma alpestris Castelnau, 1867: 112 (synonymy Sloane 1920: 157).
Phersita montana , Sloane, 1920: 156.
Theprisa montana , Moore, 1963b: 285.

#### Types.

For *Drimostomamontana*, lectotype (MGDG), labeled: Dandenong // montana / Cast. (blue label) // TYPUS (red label) // LECTOTYPUS / Drimostoma / montana / Castelnau, 1867 (black-margined red label) // MUSEO GENOVA /Coll. Castelnau (Straneo 1941). For *Drimostomaalpestris* lectotype (MGDG), triangular platen mounted female // Drimost. Alpestris / Cast. // typus (red label) // LECTOTYPUS / Drimostoma / alpestris / Castelnau, 1867 (black-margined red label) // MUSEO GENOVA / Coll. Castelnau (Straneo 1941). Both types have the pronotal lateral margins distinctly sinuate anterad the hind angles, supporting Sloane's, (1920) synonymy of these names. Castelnau (1868: 198) lists the locality of *D.montana* as “Mountains of Dandenong, Victoria”, and the locality of *D.alpestris* as “Mountains of Victoria” (Castelnau 1868: 199); these localities designated as type localities by Straneo's, (1941) lectotype designations.

#### Extended diagnosis

(n = 7). This is a large-bodied species, standardized body length 7.0–8.9 mm, with broadly based subparallel elytra, HuW/MEW = 0.67, and a cordate pronotum; the pronotal lateral margins sinuate before the hind angles (Fig. 1C). The eyes are larger than in *T.australis*, with ocular ratio 1.42–1.50, and 21–24 ommatidia crossed by a horizontal line bisecting the eye. The elytral striae are broad, smooth to slightly wavering along their length, and the discal elytral intervals are broadly convex. The pronotal basal marginal bead is continuous, with the margins posterad the laterobasal depressions only slightly angled forward relative to the median base. The pronotal median base bears 12–14 punctures each side isolated in a glossy surface, and the laterobasal depression is broadly, slightly tuberculate inside the broad lateral marginal depression just mesad the distinct, narrowly upraised lateral marginal bead. The body is dark, with piceous head capsule and pronotum, slightly paler rufo-piceous elytra, piceous ventral surface with only the proepipleuron, elytral epipleuron, and femora dark rufous. The cuticular surface is glossy, with the vertex glossy with indistinct transverse lines in irregular wrinkles across the surface, the pronotal disc with shallow transverse mesh microsculpture, sculpticells elongate, breadth 2–4× length, and the elytra subiridescent, the surface covered with a fine, elongate transverse mesh. Apical abdominal ventrite of male with two setae each side along margin, female apical ventrite with two setae each side plus a median pair of subapical setae.

***Male genitalia*** (n = 5). Aedeagal median lobe moderately robust, elongate, base broadly open on right side, basal margin heavily sclerotized dorsad basal opening (Fig. 3C); median lobe apex rounded apically, but ventral margin straight to slightly recurved in apical half of length, tip extended only slightly beyond ostium, lateral surfaces of apex densely covered with large pits; internal sac bearing a dense field of microtrichia visible in uneverted specimens (Fig. 3C), and covering much of right side of internal sac ventrad flagellar complex (Fig. 4C); flagellum elongate, robust, with a broad base; right paramere elongate, evenly narrowed in apical half to rounded tip (Fig. 5C), bearing 6–17 short setae along ventral margin in apical half, 4–8 setae on dorsal surface near apex, and two longer setae at apex; left paramere broadest in basal half, slightly narrowed to rounded tip, ventral margin glabrous or with a single short seta near apex, dorsal surface with 0–2 setae near apex, and apex with 1–3 short or long setae present; antecostal apodeme of abdominal segment IX rounded distally, lateral arms robust, their distal juncture very broad (Fig. 6C).

***Female reproductive tract*** (n = 2). Bursa copulatrix of vase-like configuration, vaginal area constricted relative to broader distal portion of bursa, length subequal to maximum breadth compressed under microslide cover slip (Fig. 7C); helminthoid sclerite present, broadly rounded apically, not extended beyond juncture with spermathecal duct; spermathecal duct very thin, elongate, sinuously joining bursa to spermathecal reservoir, length approximately 2.5× length of annulated spermathecal reservoir; spermathecal gland duct very thin, length half that of spermathecal reservoir which it joins at reservoir base; spermathecal gland comprising sclerotized stem plus membranous reservoir bearing numerous ductules; gonocoxa bipartite, basal gonocoxite 1 with single apical fringe seta, median surface glabrous, membranous ramus present (Fig. 8C); apical gonocoxite 2 narrowly subtriangular, base little extended laterally, lateral margin nearly straight basally adjacent to elongate lateral ensiform setae, apex narrowly rounded; two lateral ensiform setae and one dorsal ensiform present; two apical nematiform setae set in fossa at apical 1/4 of apical gonocoxite length.

#### Distribution and habitat.

*Theprisamontana* ranges in Victoria from the Dandenong Mountains, southeast to Gunyah and Tarra Valley (Fig. 9). Philip Darlington and family recorded specimens on Mt. Donna Buang at elevations ranging 450–1200 m (MCZ). One specimen was sifted from litter along a stream in *Nothofagus* forest at 1000 m elevation on Mt. Bullfight in the Yarra Ranges (ZMUC).

### Theprisa australis

Taxon classificationAnimaliaColeopteraCarabidae

(Castelnau)

95F9D468-1695-5537-BADB-471CC1F34045

Figures 2A , 3D , 4D , 5D , 6D , 7D , 8D , 10

Drimostoma australis Castelnau, 1867: 112.
Theprisa australis Moore, 1963b: 285.

#### Types.

***Lectotype*** male (MGDG): triangular platen-mounted male (aedeagus partly everted) // Drimost. / australis / Cast. // TYPUS (red label) // Montagne / albaiensis / & Victoria // LECTOTYPUS / Drimostoma / australis / Castelnau, 1867 (red label) // MUSEO GENOVA / Coll. Castelnau. Castelnau (1868: 198) cites the locality of this species as “Mountains of Victoria”; that locality designated type locality by Straneo (1941).

#### Extended diagnosis

(n = 5). The nearly straight, completely margined pronotal basal margin, coupled with the obtusely angulate hind angles with the pronotal lateral margins straight anterad the angles, distinguish this species from other *Theprisa* (Fig. 2A). The pronotal base is broadly punctate, with 12–13 punctures each side of the median base, the punctate surface extended across the laterobasal depression to the hind angle. The elytra are moderately constricted basally, HuW/MEW = 0.64, and the elytral disc is moderately convex, the scutellum much less depressed relative to the disc than in *T.convexa* (Fig. 2). As in *T.montana* and *T.darlingtoni*, the elytral striae are smooth, only slightly wavering along their length. But individuals of this species are nearly always smaller, with standardized body length = 5.7–7.1 mm. The eyes are smaller than in *T.montana*, with ocular ratio 1.38–1.43, 15–17 ommatidia bisected by a line horizontally crossing the eye; and little convex, with the EyL/EyD ratio ranging 3.2–4.2. Body coloration is dark, with the piceous dorsal and ventral surfaces accompanied only by paler dark rufous pro- and elytral epipleura, and rufous femora. Cuticular microsculpture is well developed, with the vertex covered with an evident isodiametric mesh, and the pronotal and elytral discs covered with dense transverse lines resulting in a cyaneous iridescence. Apical abdominal ventrite of male bearing a single seta each side along margin, female apical ventrite with two setae each side plus a median group of 3–5 setae in two subapical rows.

***Male genitalia*** (n = 5). Aedeagal median lobe robust, base broadly open on right side, basal margin sclerotized dorsad basal opening (Fig. 3D); median lobe apex broadly rounded apically, ventral margin evenly curved to meet nearly straight apical face, tip not extended beyond ostium, lateral surfaces of apex densely covered with large pits; internal sac bearing a dark field of microtrichia visible in uneverted specimens (e.g., Fig. 3D), and covering much of right side of internal sac ventrad flagellar complex (Fig. 4D); flagellum elongate, slender, with a broad base well evident in uneverted specimens (Fig. 3D); right paramere slender, evenly curved basally and narrowly extended apically to tightly rounded tip (Fig. 5D), ventral surface lined with 13–23 setae in apical 3/4 of length, 1–3 setae on dorsal surface near apex, and apex glabrous or bearing a single seta; left paramere broadest in basal half, evenly narrowed to rounded tip, ventral margin glabrous or with a single short seta near apex, dorsal surface with 0–2 setae near apex, and apex glabrous; antecostal apodeme of abdominal segment IX angled distally, lateral arms gracile, their distal juncture only slightly broader than adjoining portions of lateral arms (Fig. 6D).

***Female reproductive tract*** (n = 2). Bursa copulatrix columnar, length 1.25× maximum breadth compressed under microslide cover slip, vagina translucent, as broad as apical portion (Fig. 7D); helminthoid sclerite present, rounded apically, not extended beyond juncture with spermathecal duct; spermathecal duct stout, straight, length subequal to length of annulated spermathecal reservoir; spermathecal gland duct very thin, length twice that of spermathecal reservoir which it joins at reservoir base; spermathecal gland comprising sclerotized stem plus membranous reservoir bearing numerous ductules; gonocoxa bipartite, basal gonocoxite 1 with single, small apical fringe seta, median surface glabrous, membranous ramus present (Fig. 8D); apical gonocoxite 2 with base extended laterally, lateral margin arcuate, apex broadly rounded; two lateral ensiform setae and one dorsal ensiform present; two apical nematiform setae set in fossa at apical 1/4 of apical gonocoxite length.

#### Distribution and habitat.

**Figure 10.**
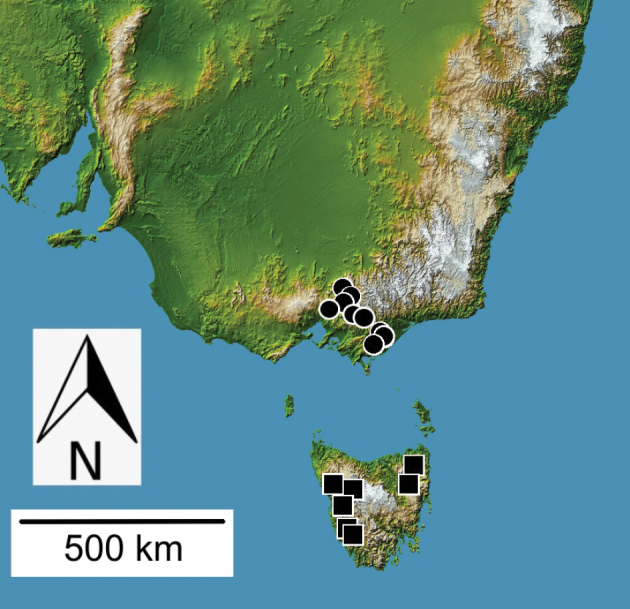
Distributional records for *Theprisa* spp.: *T.australis* (circle); *T.darlingtoni* (square).

*Theprisaaustralis* is broadly sympatric with *T.montana* in the mountains of Victoria east and southeast of Melbourne (Fig. 10). Localities where *T.australis* and *T.montana* have been collected syntopically include Gunyah, Mt. Baw Baw, Mt. Donna Buang, Sherbrooke Forest, Tarra Valley in Tarra-Bulga N. P., and Warburton. Individuals have been found in leaf and log litter associated with *Eucalyptusregnans*, *E.delegatensis* R. T. Baker, *Nothofaguscunninghamii*, and *Blechnum* ferns, and via application of pyrethrin fog insecticide to logs with fungal outgrowths (FMNH).

### Theprisa darlingtoni

Taxon classificationAnimaliaColeopteraCarabidae

Liebherr & Porch sp. nov.

A497B6DA-8CCC-5CA4-BB4E-3B6877792F9C

http://zoobank.org/21A459AB-CCDF-458F-9DB7-F786BBCAEE41

Figures 2B , 3E , 4E , 5E , 6E , 8E , 10 , 11

#### Holotype

♂ (ANIC deposition of MCZ specimen): N. of Zeehan / Jan. ’57 Tas / Darlingtons // MCZ ENT 00731903 // HOLOTYPE ♂ / Theprisa / darlingtoni / J.K. Liebherr & N. Porch 2020 (red-margined black label). Field data for the type locality include: low, wet sclerophyll forest, rain forest in places (but much cleared); Jan 17–19, 1957; usual ground methods (under logs or stones, alongside water, or drowning leaf litter in water); mostly circa 8 miles north, and along Rosebery Rd. (Darlington 1960: 125).

#### Allotypic paratype

♀. Same label and deposition as holotype.

#### Paratypes.

Same label as holotype (MCZ, 8); Tasmania: Mt. Ben Lomond [sclerophyll forest, wet gullies; logs, stones, waterside, drowning, under bark], 900–1200 m, [5-10]-iii-1957, Darlingtons(MCZ, 1); Corinna [temperate rain forest incl. *Nothofagus*; logs, stones, waterside, drowning; low elevation], [14-17]-iii-1957, Darlingtons (MCZ, 5); Whyte R., Corinna Rd. [sclerophyll forest, temperate rain forest incl. *Nothofagus*; logs, stones, waterside, drowning; low elevation], [17-18]-iii-1957, Darlingtons (MCZ, 4); Blue Tier, NE Tasmania [temperate rain forest incl. *Nothofagus*, partly cleared; logs, stones, waterside, much drowning debris], ~ 600 m, [26-31]-iii-1957, Darlingtons (MCZ, 1); Gordon R. Gorge, btw. Sir John Falls & Pyramid Is., 42.5749°S, 145.7169°E, 3-ii-1979, Howard (TMAG, 1); Frankland Range, 42.92°S, 146.01°E, 1-i-1991, Mesibov (TMAG, 1); Corinna, NW Tasmania, 4-xii-1999, Bouffard (CMNH, 1).

#### Diagnosis

(n = 5). *T.darlingtoni* is most similar to *T.montana* based on large body size; 7.4–8.0 mm versus 7.0–8.9 mm for *T.montana*. In both species the elytra are broad basally, here HuW/MEW = 0.66–0.67, with the lateral margins subparallel (Figs 1C, 2B), and the elytral striae smooth. But *T.darlingtoni* exhibits a much more pronounced tubercle laterad the deep, arcuate depression bordering the pronotal median base, and the pronotal lateral margins are only briefly sinuate laterad the basal pronotal seta (Fig. 2B), not concavely curved laterad the entire laterobasal depression as in *T.montana* (Fig. 1C). The elytral lateral marginal depression is also much broader in *T.darlingtoni*, with the rufous coloration of the depression contrasted to the rufopiceous interval 8, whereas the narrow lateral depression of *T.montana* is only slightly paler than interval 8. Cuticular microsculpture is well developed, with the vertex covered by a shallow but evident transverse mesh, the pronotal disc bearing a shallow transverse mesh, sculpticell breadth 4× length, and the elytral surface with silvery subiridescence due to the presence of dense transverse lines across the convex elytral intervals. The apical abdominal ventrite of both male and female bear three seta each side along the margin, the median pair in line with the outer two bilateral pairs.

#### Description.

***Head*** robust, ocular lobe protruded, its juncture with gena obtuse; eye diameter small relative to elongate head capsule, surface not projected beyond curve of ocular lobe, EyL/OLL = 0.69–0.71, but ocular ratio moderate, 1.40–1.46, and horizontal line across eye bisecting 21–25 ommatidia; antennal scape robust, diameter 1.4× apical diameter of pedicel; antennomeres 2 and 3 glabrous except for one dorsal seta on the pedicel and apical ring of setae on 3; antennae elongate, antennomere 9 maximal breadth 2.14× length; frontal groove broad, deep, the surface of impression irregularly strigose, the two grooves laterally arcuate, defining an ovoid raised area on frons, divergent from frontoclypeal suture to lateral margin of clypeus; broadly, deeply emarginate apical margin of labrum lined with six (rarely seven) setae, six smaller setae visible along anterolateral labral margin; mentum tooth narrowly rounded apically, sides acutely divergent; maxillary stipes trisetose, the three setae on the base in either a triangle with apex upward, or in an irregular horizontal line; ligula truncate apically, narrowed basally, trumpet shaped, its two apical setae separated by three setal diameters; paraglossae elongate, total length 3× distance from paraglossal base to ligular apical margin. ***Pronotum*** moderately transverse, lateral margins straight to slightly concave anterad acutely projected margin at basal pronotal seta; basal margin smoothly, convexly curved across width, the unmargined median base not projected beyond curve defined by distinct lateral margins posterad laterobasal depressions; median base depressed below disc, smooth basally, an arcuate line of isolated punctures each side along juncture with disc; longitudinal depression consisting of medial, deep, arcuate impression and a nearly smooth lateral tubercle, a few small punctures laterad tubercle near lateral marginal depression inside hind angles; median longitudinal impression present on median base as a series of isolated punctures, on disc consisting of lenticular depression at median base-disc juncture, and an irregular, deeply incised impression on disc; anterior transverse impression obsolete, very shallow, the median longitudinal impression continued anterad halfway across flat anterior collar of pronotum; pronotal anterior margin smooth medially, a narrow marginal bead increasingly more well developed in outer 2/3 of width each side; front angles protruded, tightly rounded; lateral marginal depression moderately narrow, broad enough so that cuticular sculpticells visible mesad beaded, raised margin; margin of lateral pronotal seta articulatory socket adjoining lateral marginal depression. ***Prosternum*** deeply canaliculate from prosternal process halfway to anterior prosternal margin, smooth over much of surface but with a few punctures or strigae at proepisternal suture anterad coxal cavity; proepisternum smooth, sutural groove with proepimeron smooth and deep. ***Elytra*** broadly ellipsoid, MEW/EL = 0.75–0.78, moderately convex, sides meeting lateral marginal depression nearly vertically, disc between striae 4 flat at midlength; basal groove arcuate, pitted at bases of striae 1–5, a small acute tooth present at juncture of groove and lateral marginal depression; stria 8 very deep and continuous between anterior and posterior series of lateral setae; apical carina of interval 8 narrowly upraised along stria 7, interval 8 a vertical lateral carina there; subapical sinuation abrupt, the internal plica visible ventrad deepest part of sinuation. ***Mesepisternum*** broadly punctate, ~ 19–22 punctures in 3–4 confused vertical rows. ***Metepisternum*** trapezoidal, maximum width subequal to lateral length; metepisternal-metepimeral suture complete. ***Legs*** gracile, the femora elongate and meso- and metatibiae only slightly broadened in apical half; metatarsomere 1 moderately elongate, length 0.20× tibial length, lateral sulci present on mesal and lateral faces just dorsad the ventrolateral setae. ***Abdominal ventrites*** 2–6 smooth laterally, but hind margins of ventrites 2–5 concavely sinuate laterally, the sutures deeper in association with sinuation; suture between ventrites 1 and 2 deep, slightly curved anteriorly at midlength; suture between ventrites 2 and 3 complete laterally.

***Male genitalia*** (n = 5). Aedeagal median lobe elongate, moderately robust, base broadly open on right side, basal margin heavily sclerotized dorsad basal opening (Fig. 3E); apical face joined to slightly arcuate ventral margin at right angle, flattened tip not extended beyond ostium, lateral surfaces of apex smooth or with only several indistinct pits; internal sac largely membranous, with only very elongate flagellum and associated basal articulatory sclerites darker (Figs 3E, 4E); right paramere elongate, slightly angled at midlength, apex narrowly rounded, slightly asymmetrical in outside lateral view (Fig. 5E), bearing 10–19 short and long setae along ventral margin in apical half, 2–4 setae on dorsal surface near apex, two or three setae at apex; left paramere broadest in basal half, narrowed in apical half but broadened apically to broadly rounded tip, ventral margin with 3–7 setae near apex, dorsal surface glabrous or with a single seta, and apex with two or three longer setae; antecostal apodeme of abdominal segment IX angulate distally, lateral arms gracile, their distal juncture only slightly broadened (Fig. 6E).

**Figure 11.**
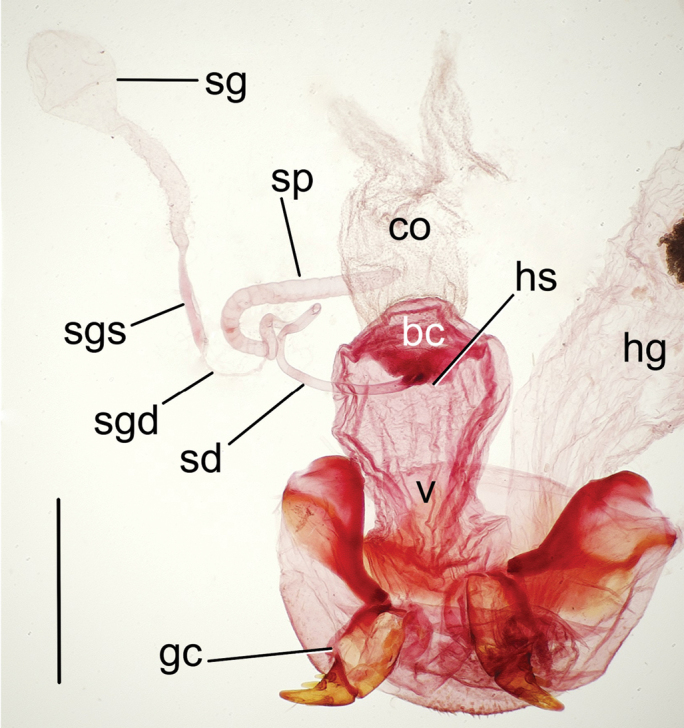
Female reproductive tract of *Theprisadarlingtoni*, ventral view; scale bar = 0.50 mm. Abbreviations: bc, bursa copulatrix; co, common oviduct; gc, gonocoxa; hg, hindgut; hs, helminthoid sclerite; sd, spermathecal duct; sg, spermathecal gland; sgd, spermathecal gland duct; sgs, spermathecal gland stem; sp, spermatheca; v, vagina.

***Female reproductive tract*** (n = 1). Bursa copulatrix columnar, length 1.5× maximum breadth compressed under microslide cover slip, vagina translucent, apical portion of bursa staining more darkly with Chlorazol black (Fig. 11); helminthoid sclerite present, broadly rounded apically, not extended beyond juncture with spermathecal duct; spermathecal duct thick, sclerotized, coiled sinuously to spermatheca, length twice that of annulated spermathecal reservoir; spermathecal gland duct very thin, length half that of spermathecal reservoir which it joins at reservoir base; spermathecal gland comprising sclerotized stem plus membranous reservoir bearing numerous ductules; gonocoxa bipartite, basal gonocoxite 1 with single apical fringe seta, median surface glabrous, membranous ramus present (Fig. 8E); apical gonocoxite 2 with base extended laterally, lateral margin arcuate, apex narrowly rounded; two lateral ensiform setae and one dorsal ensiform seta present; two apical nematiform setae set in fossa in apical 1/4 of apical gonocoxite length.

#### Etymology.

This species honors Professor Philip J. Darlington, Jr., who among his many roles, systematically collected carabid beetles across Australia, and served as the first post-doctoral supervisor for Dr. Terry L. Erwin (Kavanaugh 2020). Terry championed a Darlingtonian world view regarding the diversification of carabid beetles (Erwin 1981), never losing sight of those questions that tied both men to primary observations made over long careers spent in the field.

#### Distribution and habitat.

*Theprisadarlingtoni* is known from localities spanning northern and western Tasmania, from Blue Tier on the east to Corinna in the northwest, to the Frankland Range in southwest Tasmania (Fig. 10). Collecting localities lie within the Ben Lomond, Central Highlands, and West biogeographic regions (IBRA 2004). It has been collected syntopically with *T.convexa* at three localities: Corinna (Bouffard, CMNH), Whyte River at Corinna Road (Darlingtons, MCZ), and Zeehan (Simson, SAMA, and Darlingtons, MCZ), although these respective 1891 and 1957 collections were separated by 66 years. The five sites at which the Darlington family collected this species (Blue Tier, Corinna and nearby Whyte River, Mt. Ben Lomond, and Zeehan) were reported to support sclerophyll forest, wet sclerophyll forest, temperate rain forest with *Nothofagus*, and wet gullies, with specimens at all localities collected under logs and stones, along water sources, and via immersion of leaf litter in standing water (Darlington 1960).

##### Phylogenetic analysis

**Figure 12.**
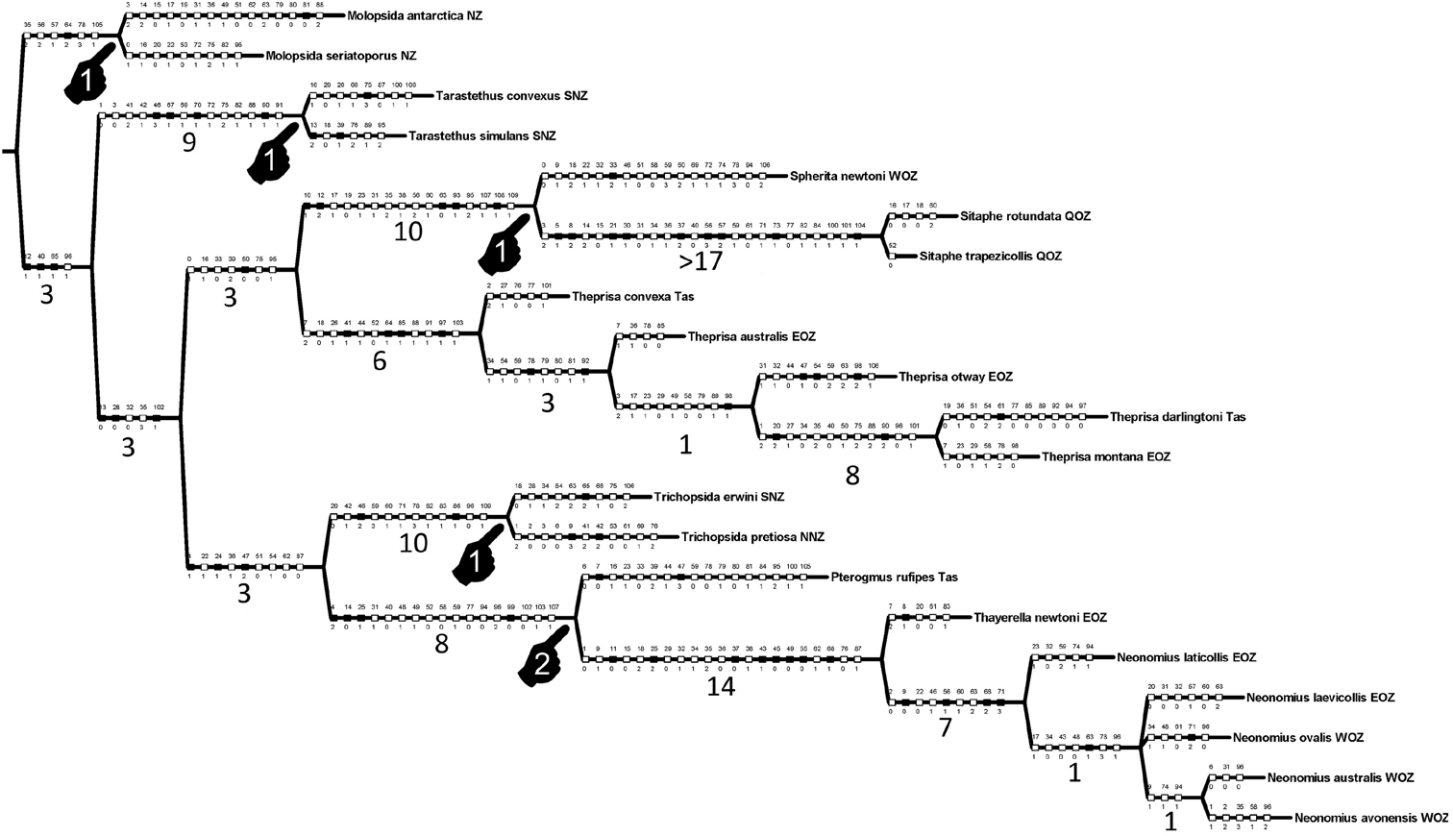
Strict consensus of two equally parsimonious, 380-step cladograms including *Theprisa* spp. and cladistically neighboring moriomorphine taxa of the subtribe Tropopterina (see text); consensus cladogram length 382 steps, CI = 0.43, RI = 0.66. Character numbers are shown above cladogram edges, character states below. Cladogram root placed so tree topology is compatible with the more inclusive cladogram of Liebherr (2020: fig. 1). Filled squares represent unique state transformations on cladogram, open squares indicate state transformations that occur more than once on cladogram. Clades of this analysis previously recovered in Liebherr (2020) are indicated by pointers numbered 1; the clade subtended by *Pterogmus* reported in Liebherr (2019) indicated by the pointer numbered 2. Decay indices (i.e., Bremer Support values) shown beneath cladogram edges for all internal edges of the cladogram. Geographic distributions of species are indicated by abbreviations following species epithets: **EOZ**, southeastern Australia; i.e., A. C. T., New South Wales and Victoria; **NNZ**, North Island, New Zealand; **NZ**, New Zealand; **SNZ**, South Island, New Zealand; **QOZ**, Queensland; **Tas**, Tasmania; **WOZ**, Western Australia

Parsimony analysis finds two cladograms of 380 steps, with the strict consensus collapsing one node within representatives of *Neonomius* (Fig. 12). Monophyly of the five *Theprisa* spp. is recovered and this clade is adelphotaxon to a clade comprising *Spherita* and *Sitaphe*. This sister-group relationship was not recovered in Liebherr (2020), but that analysis was comprehensive taxonomically, and this less so as it was focused only on demonstrating monophyly of *Theprisa*. In the current analysis, there is also very high support for the adelphotaxon relationship of *Sitaphe* + *Spherita* (Fig. 12). There is substantial support within the morphological character data for *Theprisa* monophyly, with the genus obtaining a decay index value of 6. Several characters unambiguously support *Theprisa* monophyly. The uniquely pitted aedeagal apex observed in four of the five species (character 97, state 1; Fig. 3A–D) has not been observed in any other moriomorphine. The prosternum is grooved laterally about halfway along its length each side near the proepisternum (character 41, state 1), a condition not observed in the included taxa except for New Zealand's, *Tarastethus* spp. and *Trichopsidapretiosa* (Broun), though those taxa exhibit a series of pits in this position. The metepisternum and metepimeron are fused laterally (character 64, state 1), no doubt in association with the extremely reduced metathoracic flight-wing apparatus. And the eyes are small, with the ocular lobe ratio (EyL/OLL) less than 0.72 (character 18, state 0), though *T.montana* exhibits variation in this character with some individuals having larger eyes. Finally, the spermathecal gland has a well-sclerotized stem (Figs 7, 11; character 103, state 1), a condition evolved independently in the clade subtended by *Pterogmus* (Fig. 12; Liebherr 2019).

## Discussion

*Theprisaotway* joins several other carabid beetle species precinctive to the Otway Ranges (Table 1). Based on biogeographic relationships among carabid beetles, area relationships of the Otways are varied, including: Tasmania alone (*Stichonotus*), southeastern Australia plus Tasmania (*Theprisa*), and eastern Victoria and New South Wales (*Eutrechus*, *Moriomorpha*, and *Pseudagonica*). Based on relationships among taxa in the *Thayerella* + *Neonomius* clade, the Otways represent a peripherally isolated sister-area to the remainder of temperate mainland Australia. Here again a trans-Bassian areal connection is supported, given the hypothesis that the Tasmanian monotypic genus *Pterogmus* Sloane is adelphotaxon to this clade (Liebherr 2019).

**Table 1.** Examples of species precinctive to the Otway Ranges, along with the aggregate distributions of closely related species. Adelphotaxa to Otway endemics may be circumscribed by a phylogenetic hypothesis (1), or defined by common membership in a generic-level or species-group taxon (2). Abbreviations of geographic areas include: WA, Western Australia; SA, South Australia; eV, eastern Victoria; NSW, New South Wales; Tas, Tasmania; NZ, New Zealand.

Order	Family	Otway endemic	Areas of Adelphotaxon						Reference
			WA	SA	eV	NSW	Tas	NZ	
Acari	Ologamasidae	*Evanssellusmedusa* (2)						x	Lee (1967)
Araneae	Archaeidae	*Zephyrarchaeaporchi* (1)		x	x				Rix and Harvey (2012)
	Gradungulidae	*Progradungulaotwayensis* (1)				x			Machalik et al. (2013)
Plecoptera	Eustheniidae	*Eusthenianothofagi* (2)			x	x			Zwick (1979)
	Notonemouridae	*Austrocercelladistans* (2)			x	x	x		Theischinger (1984)
Hemiptera	Peloridiidae	*Hemiodoecusacutus* (1)			x	x	x		Burckhardt (2009)
		*Hemiowoodwardiawilsoni* (1)			x	x	x		Burckhardt (2009)
Coleoptera	Carabidae	*Stichonotuslimbatus* (2)					x		Baehr (2012)
		*Eutrechusotwayensis* (2)			x	x			Moore (1972)
		*Thayerellanewtoni* (1)	x	x	x	x			Liebherr (2019)
		*Theprisaotway* sp. nov. (1)			x	x	x		Herein
		*Moriomorphacurvipes* (2)			x	x			Baehr (2011)
		*Pseudagonicanitida* s. s. (2)			x	x			Moore (1960, 1963)
	Leiodidae	*Nargiotesnewtoni* (2)			x				Giachino and Peck (2003)
Diptera	Keroplatidae	*Arachnocampaotwayensis* (1)			x	x	x		Baker (2008, 2010)

Zoogeographic relationships of the Otway Ranges that implicate southeastern Australia and Tasmania are also indicated for *Austrocercella* stoneflies, and *Hemiodoecus* and *Hemiowoodwardia* moss bugs, plus *Arachnocampa* glow-worms (Table 1). Geographically more restricted biogeographic relationships to taxa in South Australia and Victoria (*Zephyrarchaea* assassin spiders), New South Wales (*Progradungula* odd-clawed spiders), or eastern Victoria (*Nargiotes* small carrion beetles) add to the disparate examples of Otway Range biotic connections (Table 1). Finally, the Otway endemic mite, *Evanssellusmedusa* (Lee) (Castilho et al. 2016), represents a disjunct Gondwanan pattern. Its sister species, *E.foliatus* (Ryke), occupies New Zealand, whereas the four species of their sister group, *Heterogamasus* Ryke, are distributed in southern South America (Lee 1967). Summing over these examples, the biogeographical history of the Otways has involved autochthonous speciation fragmenting ancestral ranges that include a variety of other areas of endemism, with those events arguably occurring at different times in the past. Because the relationships to these areas involve different levels of phylogenetic divergence, it is clear that the Otway area of endemism has accrued its distinct biogeographic status through various and sundry means, and thus should be considered a mosaic area of endemism that represents products of numerous different phylogenetic and biogeographic histories.

All *Theprisa* spp. exhibit vestigial metathoracic flight wings and dorsally convex, ovoid elytra indicating a long evolutionary history of apterous ancestors. Indeed, given the phylogenetic hypothesis proposed above (Fig. 12), *Theprisa* and its adelphotaxon, the clade *Spherita* + *Sitaphe*, all comprise exclusively apterous species. As such, occupation of relatively restricted geographic ranges in montane Australia (Figs 9, 10) by species with much reduced vagility is to be expected (Darlington 1936). These attributes would place *Theprisa* as an evolutionary terminus of the taxon pulse model (Erwin 1981). This suggests that divergence of the Tasmanian and Victorian species *T.darlingtoni* and *T.montana* respectively (Fig. 12) has not involved Pleistocene-aged trans-Bassian dispersal during low sea stands of the past 60,000 years (Orchiston 1979; Lambert and Nakada 1990), but rather an older episode of vicariance implicating presently isolated montane areas. *Theprisaconvexa*, the other Tasmanian species stands as adelphotaxon to the other four species, thereby supporting a second, earlier episode of divergence between ancestral taxa occupying Tasmania and mainland Australia. Additionally, paraphyletic relationships among the New Zealand moriomorphine genera, *Molopsida* White, *Tarastethus* Sharp, *Trichopsida* Larochelle and Larivière, and *Theprisa* (Fig. 12; Liebherr 2020: fig. 1) could point to either older diversification of all of these genera via fragmentation of gondwanan terranes, or more recent dispersal events across the Tasman Sea as means to establish these biogeographical disjunctions. Mutual biogeographic paraphyly is also defined by phylogenetic relationships among ancient Australian and New Zealand cave crickets (Orthoptera, Rhaphidophoridae), with estimated divergence times of these taxa arguably consistent with Gondwanan vicariance (Beasley-Hall et al. 2018; Allegrucci and Sbordoni 2019). This contribution helps build a stable classification for Moriomorphini, setting the course for additional phylogenetic research that can test whether the similar biogeographic patterns observed among moriomorphine genera are also founded on the ancient Gondwanan fragmentation of Australia, Tasmania, and New Zealand.

## Supplementary Material

XML Treatment for Theprisa

XML Treatment for Theprisa otway

XML Treatment for Theprisa convexa

XML Treatment for Theprisa montana

XML Treatment for Theprisa australis

XML Treatment for Theprisa darlingtoni

## Supplementary materials

Supplementary material 1 Images of *Phersitaconvexa* Sloane type series to accompany Data type images Explanation note The following images document the syntype series of *Phersitaconvexa* Sloane, including the Lectotype labelled by P.J. Darlington, Jr. in 1957 plus 10 Paralectotypes. The original type series constituted 11 specimens, with one of the South Australian Museum specimens indicated as the primary type by its labelling. Darlington followed that convention, as did A.M. Lea when the types were registered in SAMA. Following is a listing of images that account for the lectotype and the 10 paralectotypes. The paralectotype numbering follows the sequential listing in Liebherr et al. (2021). https://binary.pensoft.net/file/553862This dataset is made available under the Open Database License (http://opendatacommons.org/licenses/odbl/1.0/). The Open Database License (ODbL) is a license agreement intended to allow users to freely share, modify, and use this Dataset while maintaining this same freedom for others, provided that the original source and author(s) are credited.James K. Liebherr, Nick Porch, Matthew Shaw, Bronte E. Sinclair, David R. Maddison

Supplementary material 2 Data file used for cladistic analysis of *Theprisa* and closely related moriomorphine genera Data type phylogenetic tree Explanation note .ss file in NONA format (Goloboff 1999) output from Winclada (Nixon 2002) (110 characters, 16 taxa, 2 most parsimonious trees and strict consensus tree). https://binary.pensoft.net/file/553863This dataset is made available under the Open Database License (http://opendatacommons.org/licenses/odbl/1.0/). The Open Database License (ODbL) is a license agreement intended to allow users to freely share, modify, and use this Dataset while maintaining this same freedom for others, provided that the original source and author(s) are credited.James K. Liebherr, Nick Porch, Matthew Shaw, Bronte E. Sinclair, David R. Maddison
