# Supplementary material for: Systematic revision of the trans-Bassian moriomorphine genus Theprisa Moore (Coleoptera, Carabidae)
Source: Zookeys. 2021 Jun 16;1044:339–73. doi: 10.3897/zookeys.1044.62335 (PMC8390884; doi:10.3897/zookeys.1044.62335)
Supplement: Supplementary material 1 — Images of Phersitaconvexa Sloane type series to accompany [file zookeys-1044-339-s001.pdf]

Supplementary Material 1

Images of *Phersita convexa* Sloane type series to accompany

Systematic revision of the trans-Bassian moriomorphine genus *Theprisa* Moore (Coleoptera: Carabidae)

James K. Liebherr, Nick Porch, Matthew Shaw, Bronte E. Sinclair and David R. Maddison

Photos courtesy of B. E. Sinclair (ANIC) and M. Shaw (SAMA)

The following images document the syntype series of *Phersita convexa* Sloane, including the Lectotype labelled by P.J. Darlington, Jr. in 1957 plus 10 Paralectotypes. The original type series constituted 11 specimens, with one of the South Australian Museum specimens indicated as the primary type by its labelling. Darlington followed that convention, as did A.M. Lea when the types were registered in SAMA. Following is a listing of images that account for the lectotype and the 10 paralectotypes. The paralectotype numbering follows the sequential listing in Liebherr et al. (2021).

1. Register of listed images (this page).

2. Paralectotypes 1 & 2 (ANIC) in situ on mounting card.

3. Paralectotype 1 dorsal view.

4. Paralectotype 2 dorsal view.

5. Paralectotypes 1 & 2 specimen labels. Paratype label added by P.J. Darlington, Jr. (T. Weir, pers. comm.)

6. Paralectotype 3 (ANIC), dorsal view.

7. Paralectotype 3 labels, upper side. Paratype label added by P.J. Darlington, Jr. (T. Weir, pers. comm.)

8. Paralectotype 3 labels, obverse side of Zeehan locality label.

9. Lectotype male (SAMA), dorsal view.

10. Lectotype male, lateral view.

11. Lectotype male labels. “Lectoholo” label of PJD (Philip J. Darlington, Jr.) treated as lectotype label, with lectotype designated in Liebherr et al. (2021).

12. Paralectotype 4 male (SAMA), dorsal view.

13. Paralectotype 4 labels (obverse sides blank).

14. Paralectotype 5 female (SAMA), dorsal view.

15. Paralectotype 6 female (SAMA), dorsal view.

16. Paralectotypes 5 and 6 labels.

17. Paralectotype 7 (SAMA), dorsal view.

18. Paralectotype 8 (SAMA), dorsal view.

19. Paralectotypes 7 and 8 (SAMA), locality and determination labels.

20. Paralectotypes 7 and 8 (SAMA), museum registration labels.

21. Paralectotypes 9 and 10 (SAMA) in situ on mounting card, with locality and determination labels.

22. Paralectotypes 9 and 10 (SAMA), syntype and museum registration labels.

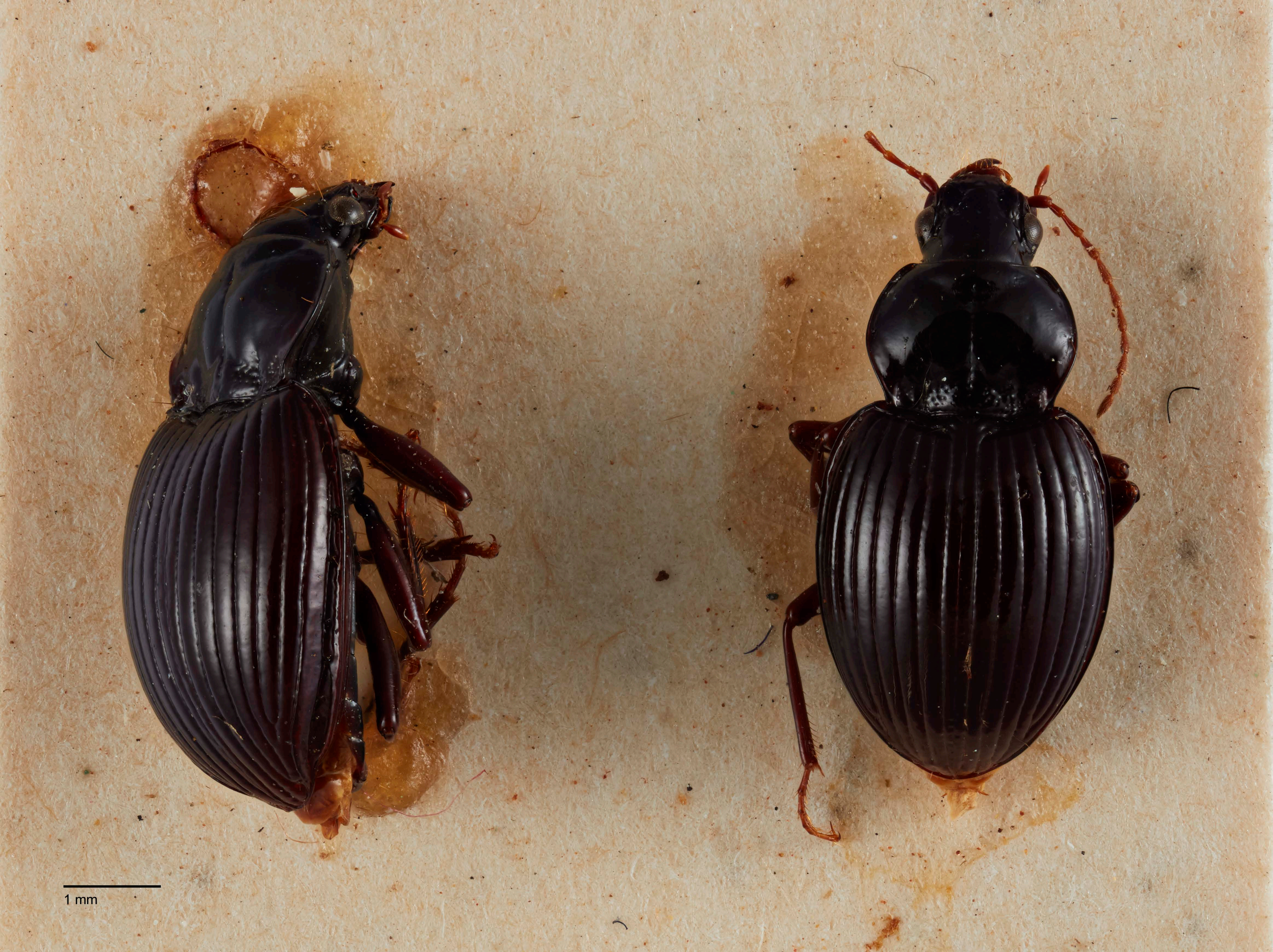
1 mm

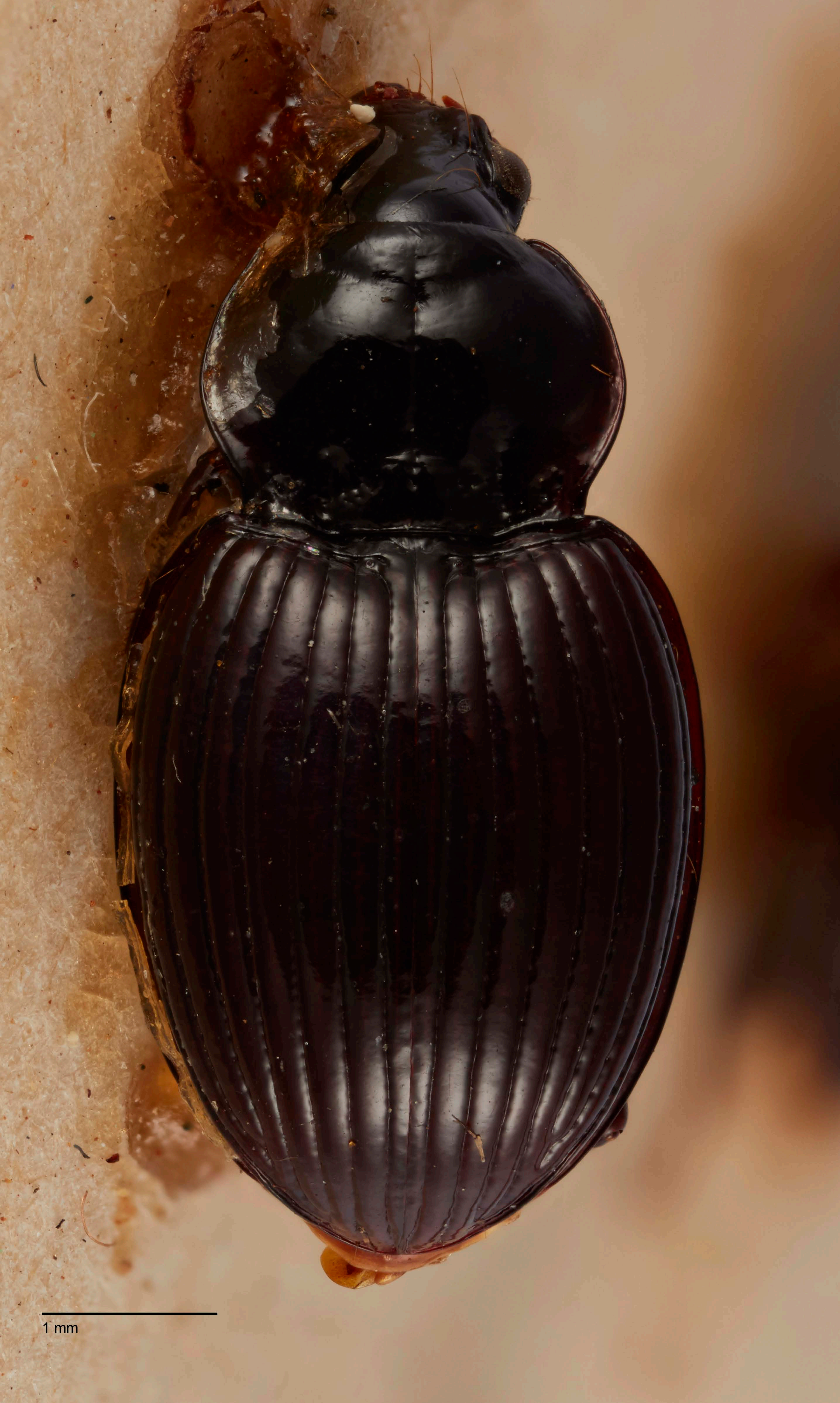
1 mm

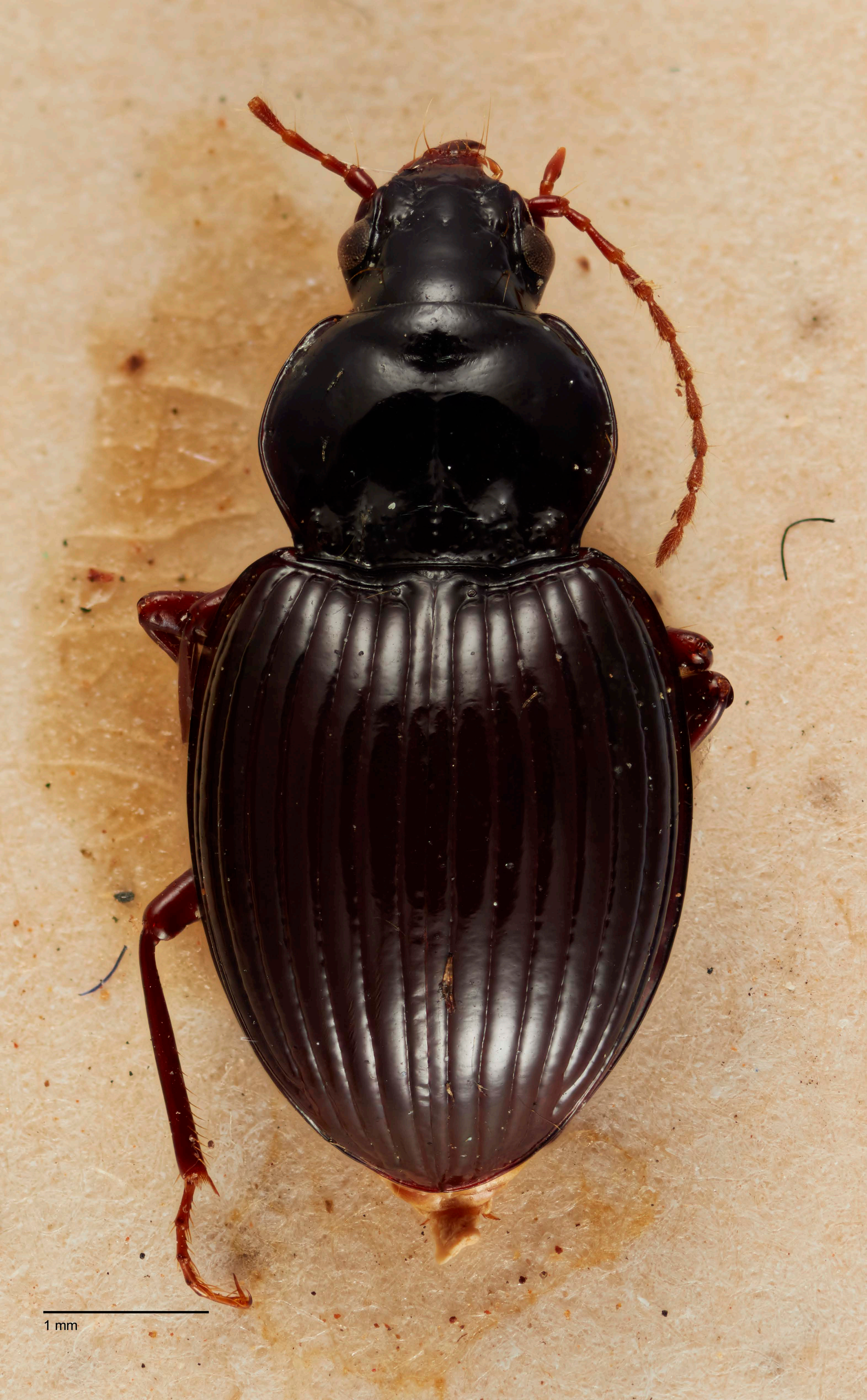
1 mm

Strahan
Tas: Lea
& Carter

Phersita
convexa Sl.
Id. by T. G. Sloane.

Co-type.

PARATYPE.

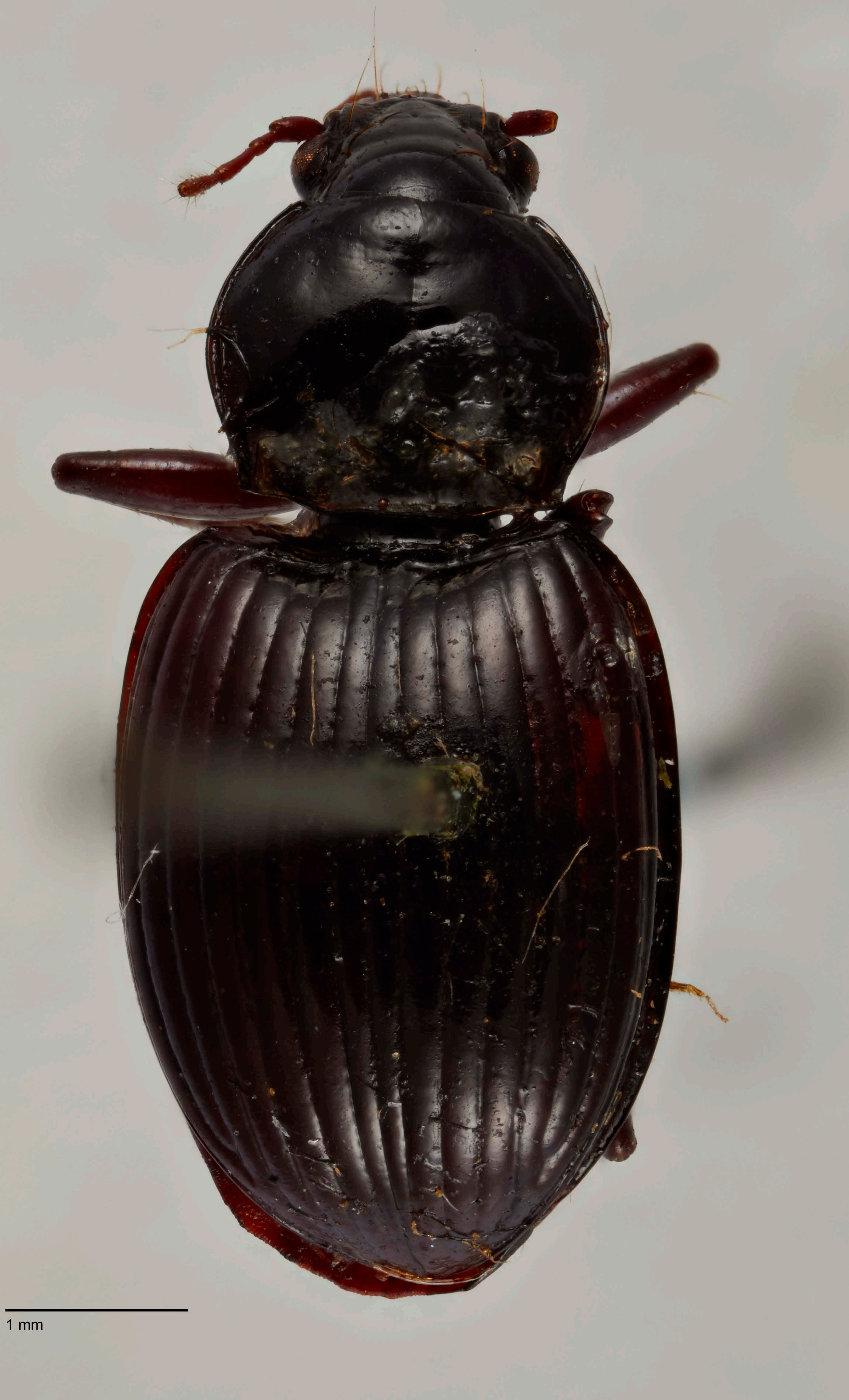
1 mm

Zeehan

Phersila
convexa Sl.
Id. by T. G. Sloane.

Co-type.

PARATYPE.

15/91.

Phersila convexa Sl.
Id. by T. G. Sloane.

Co-type.

PARATYPE.

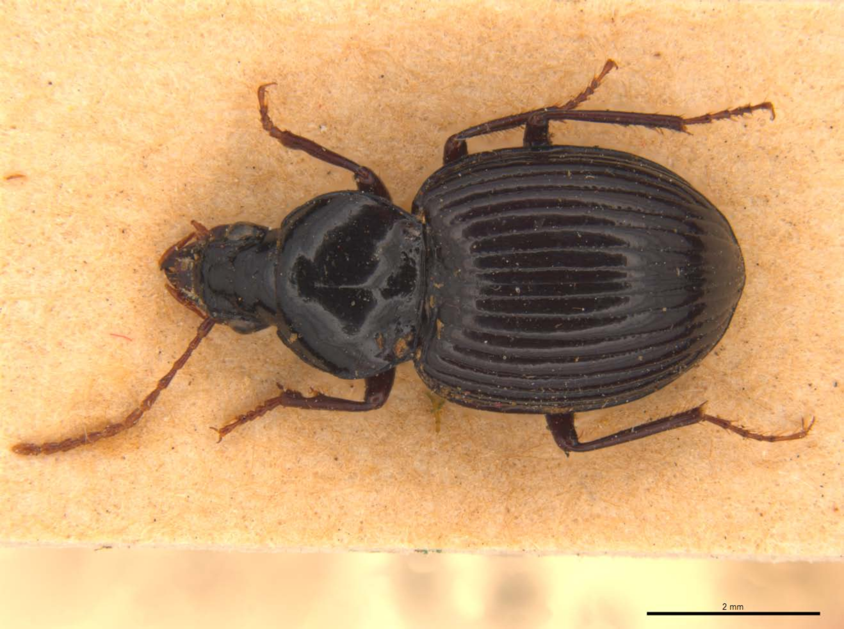
2 mm

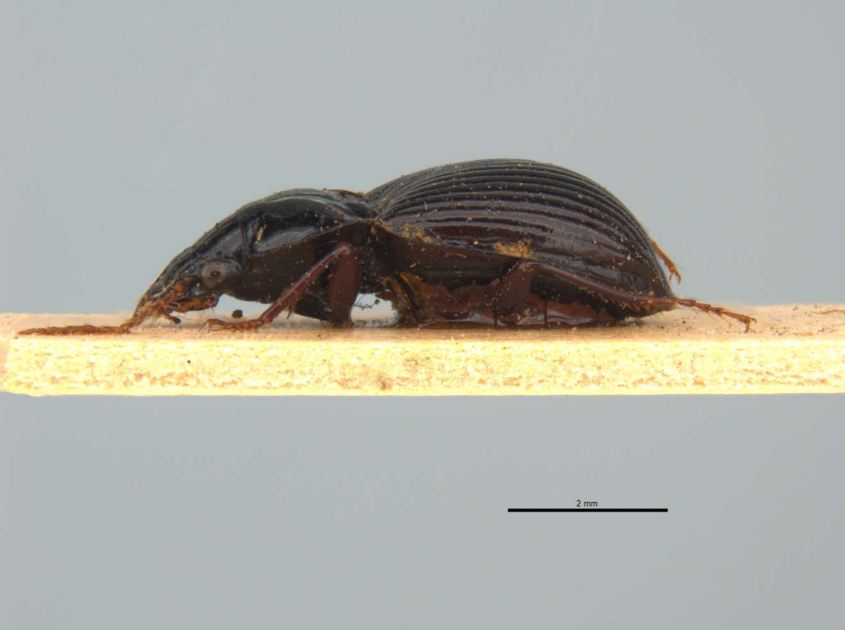
2 mm

Type.

T
Zeehan

Phersita
convexa Sl.
Id by T. G. Sloane

Lectoholo.
P. convexa
PJD Sl.

I.115-64
Phersita
convexa Sl
Tasmania
EI 71

SAMA Database
No. 25-035536

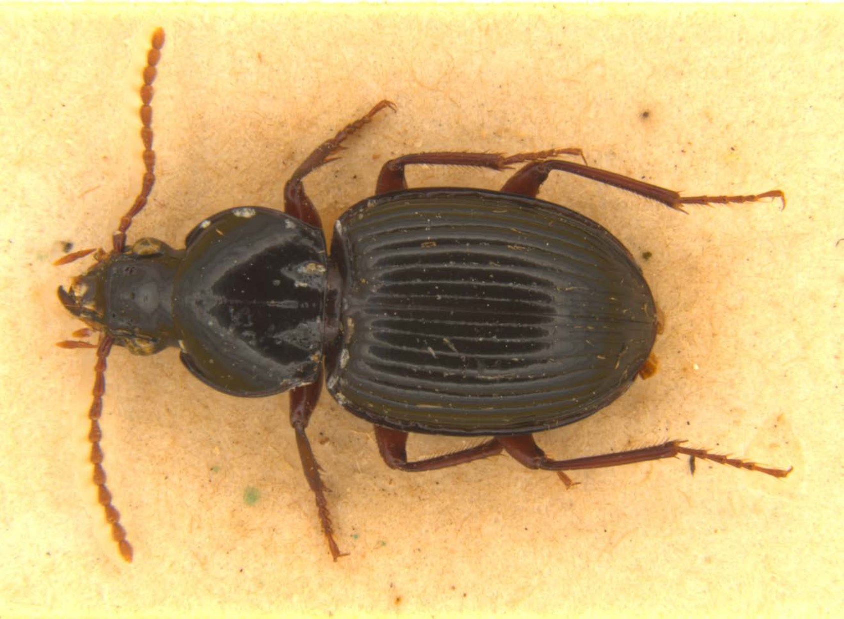

SAMA Database
No. 25-039812

Phersita
convexa Sl.
Id. by T. G. Sloane

Zeehan

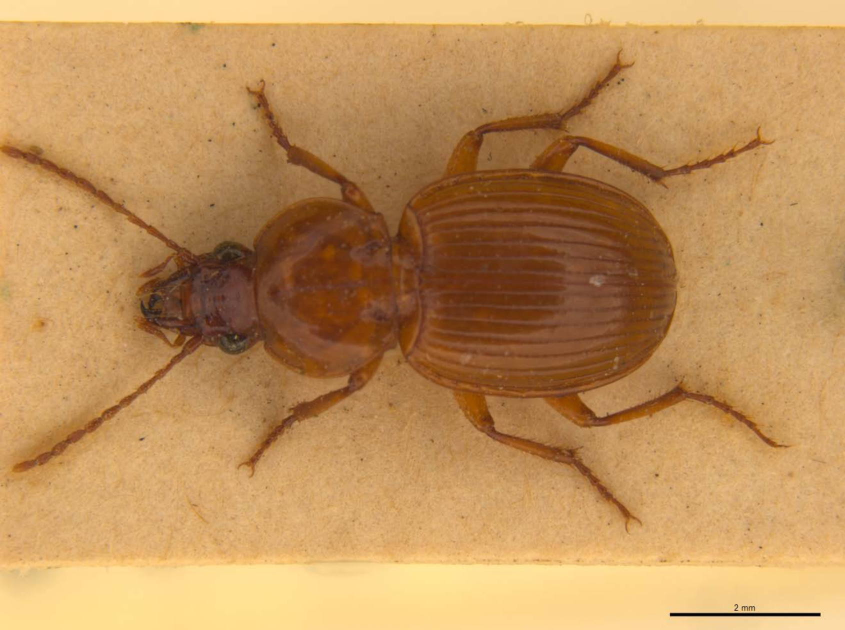
2 mm

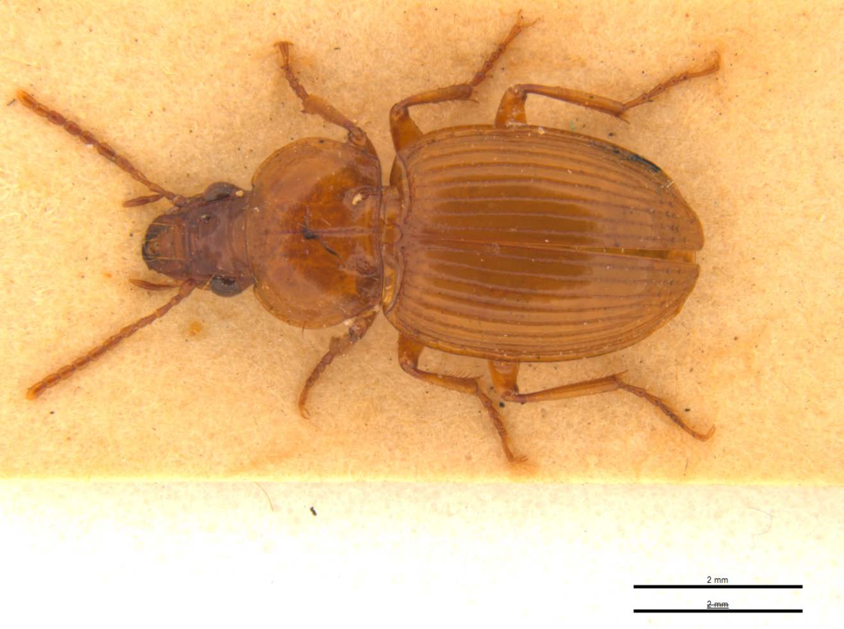
2 mm
2 mm

Phersita convexa Sl.
Id. by T. G. Sloane

3123

Tasmania
A. Simson

Tasmania
A. Simson

SAMA Database
No. 25-039813

SAMA Database
No. 25-039814

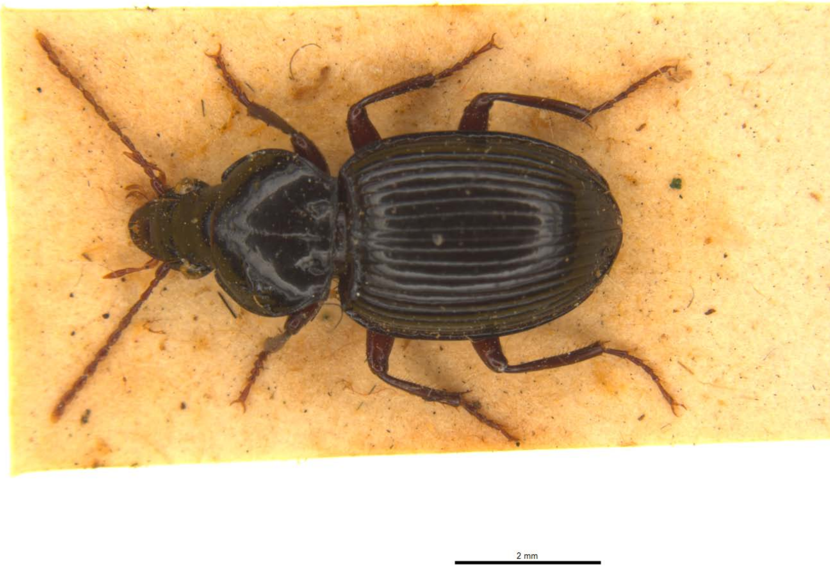
2 mm

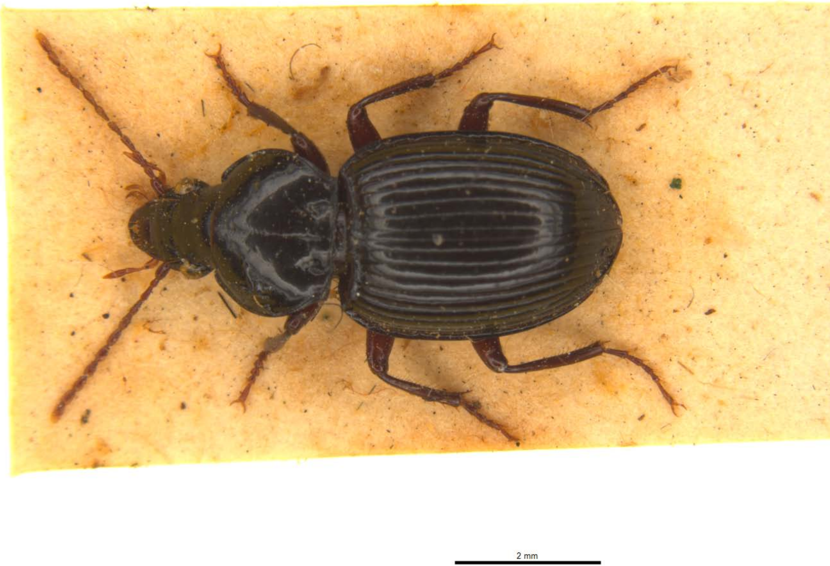
2 mm

3123

Tasmania
A. Simson

Tasmania
A. Simson

Phersita
convexa Bl.
Id. by T. G. Sloane

19594
Phersita
convexa [illegible]

[illegible]

Tasmania

SAMA Database
No. 25-039819

SAMA Database
No. 25-039820

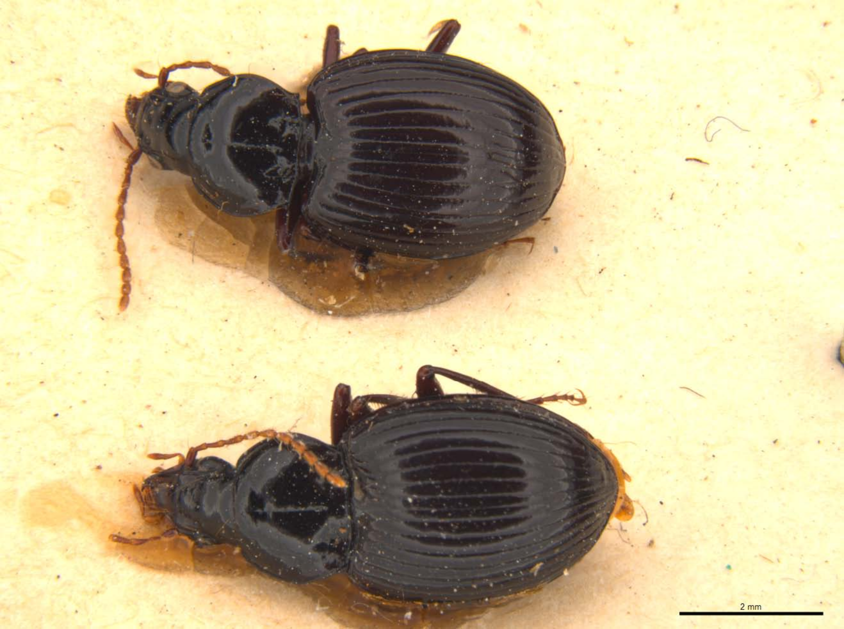
2 mm

Waratah
Tas : Lea
& Carter

Waratah
Tas : Lea
& Carter

Phersita
convexa Sl.
Id. by T. G. Sloane

Co-type.

SAMA Database
No. 25-039821

SAMA Database
No. 25-039822
